# A five-residue motif for the design of domain swapping in proteins

**DOI:** 10.1038/s41467-019-08295-x

**Published:** 2019-01-28

**Authors:** Neha Nandwani, Parag Surana, Hitendra Negi, Nahren M. Mascarenhas, Jayant B. Udgaonkar, Ranabir Das, Shachi Gosavi

**Affiliations:** 1 National Centre for Biological Sciences, Tata Institute of Fundamental Research, Bengaluru, 560065 India; 2 Sastra University, Thanjavur, 613402 India; 3 Indian Institute of Science Education and Research, Pune, 411008 India; 4 Present Address: Sacred Heart College, Tirupattur, Tamil Nadu 635601 India

## Abstract

Domain swapping is the process by which identical monomeric proteins exchange structural elements to generate dimers/oligomers. Although engineered domain swapping is a compelling strategy for protein assembly, its application has been limited due to the lack of simple and reliable design approaches. Here, we demonstrate that the hydrophobic five-residue ‘cystatin motif’ (QVVAG) from the domain-swapping protein Stefin B, when engineered into a solvent-exposed, tight surface loop between two β-strands prevents the loop from folding back upon itself, and drives domain swapping in non-domain-swapping proteins. High-resolution structural studies demonstrate that engineering the QVVAG stretch independently into various surface loops of four structurally distinct non-domain-swapping proteins enabled the design of different modes of domain swapping in these proteins, including single, double and open-ended domain swapping. These results suggest that the introduction of the QVVAG motif can be used as a mutational approach for engineering domain swapping in diverse β-hairpin proteins.

## Abstract (web summary)

Designing interfaces that can induce protein-protein interactions is a challenging problem. Here the authors show that a five amino acid sequence known to mediate domain swapping in cystatins can drive oligomerization when grafted onto functionally and structurally unrelated host proteins, providing a simple approach to the design of protein assemblies.

## Introduction

Rational design of protein–protein interactions can be used to build supramolecular assemblies capable of performing both biological and bio-inspired functions^1^. The formation of a dimer is a basic step in building such assemblies^2^, and diverse methods have been invented to engineer protein homodimers and heterodimers^3^. However, the required protein manipulation is generally difficult because of the presence of a complex array of cooperative and long-range interactions in proteins. This structural complexity and the marginal stability of proteins necessitate the optimization of each design approach in a protein-specific manner. Thus, the number of proteins and protein sites amenable to a given design strategy is typically low.

**Fig. 1:**
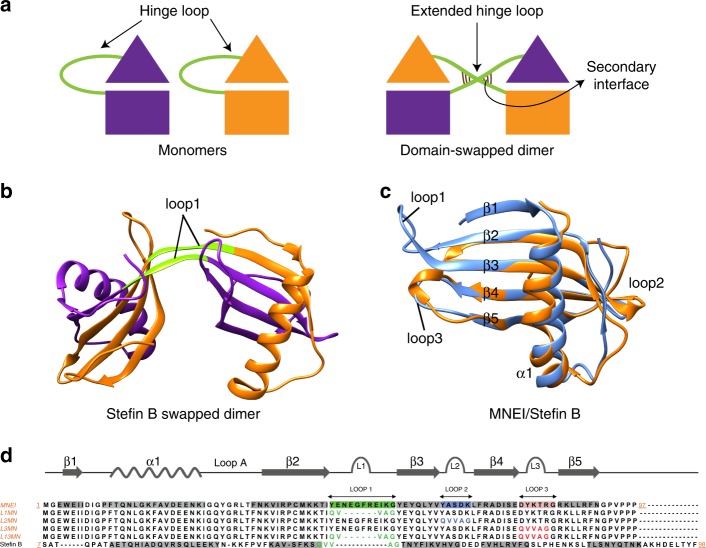
Rational design of domain swapping in MNEI. **a** Schematic representation of domain swapping. The hinge loop, connecting the exchangeable domains, adopts an extended conformation in the swapped dimer. Each monomer-like half of the swapped dimer, formed by contributions from the two polypeptide chains (or protomers), is referred to as a “functional unit”. The proximal arrangement of the polypeptide chains in the domain-swapped conformation may lead to the formation of a novel intermolecular interface (secondary interface), which is not found in the monomeric structure. The secondary interface is shown in the cartoon as a set of new interactions established between the two polypeptide chains in the region of crossover. **b** Structure of the domain-swapped dimer of stefin B (PDB ID: 2OCT) is shown. The two polypeptide chains contributing to the dimer are shown in purple and orange. Sub-domains β1-α1-loopA-β2 and β3-loop2-β4-loop3-β5 are exchanged between the two protomers. Loop1 (green) containing the QVVAG stretch acts as the hinge loop. **c** Structural superposition of MNEI (PDB ID: 1IV7, blue) and stefin B monomer (PDB ID: 4N6V, orange) is shown. **d** Structure-based sequence alignment of residues 7–98 of Stefin B with the full sequence of MNEI (1–97) is shown. The MNEI variants designed in this study are also shown in the alignment. The secondary structures are indicated above the alignment in gray. The loop sequences are colored as green: loop1, blue: loop2, and red: loop3

Several proteins dimerize (or oligomerize) naturally through domain swapping (sometimes termed 3D domain swapping^4^). In domain swapping, two protein molecules exchange “domains” or structural units connected by a hinge loop, such that intermolecular interactions replace the intramolecular interactions at the dimer interface of each monomer^4–8^ (Fig. 1a). This gives rise to a dimer containing two monomeric units which are almost identical to the monomeric protein. Domain swapping is well-suited for the construction of oligomeric interfaces for the following three reasons. First, the structural diversity of domain swapping proteins reported so far indicates that protein structure does not place strong restrictions on the design of domain swapping^9,10^. Second, domain swapping is likely to generate stable multimers because the same contacts that stabilize the protein monomer also stabilize the protein dimer. Further, several proteins domain-swap from the unfolded state^11–13^ and thus, a barrier larger than the folding free energy barrier, separates the monomer and the domain-swapped dimer. Finally, domain swapping can result in the formation of structurally complex oligomeric assemblies. Proteins can swap domains in an open-ended^4^ manner, leading to the formation of complex linear assemblies^14,15^. Proteins can also swap more than one domain^7^, either separately^16,17^, or simultaneously^18,19^ (referred to as double domain swapping^10^), leading to the formation of different protein assemblies from a single protein^10^. Moreover, nature uses domain swapping not only as a mechanism for oligomer assembly, but also to encode for novel functions and for the evolution of novel protein folds^4,20,21^.

Thus, engineered domain swapping is likely to be a simple and universal strategy for the design of protein oligomers and assemblies. A few non-natural domain-swapped proteins have been designed using ad hoc methods^11,22–33^. Such studies have collectively led to an understanding of the general principles of domain swapping design. Introduction of conformational strain in the monomer, by altering the physico-chemical properties of the putative hinge loop, is expected to drive domain swapping. The underlying driving force for oligomerization is the change in the conformation of the modified and strained hinge loop to an energetically favorable extended conformation in the domain-swapped structure^6,7,12^. However, these studies have not led to a specific mutational strategy for the introduction of domain swapping into diverse monomeric proteins. In a previous study, we noticed that placing a bulky, hydrophobic residue at the apex of a solvent-exposed, strained β-turn results in domain swapping^34^. Based on this result, we asked if engineering the largely hydrophobic pentapeptide motif present in the domain-swapping cystatin (β1-α1-β2-β3-β4-β5 topology) proteins^35^ into the β-turns of proteins could be a general strategy for designing domain swapping. In the domain-swapping cystatins, the loop connecting β2–β3 has a QXVXG consensus motif, which has been implicated in both protease inhibition and domain swapping^35–39^ (Fig. 1b).

Here, we engineer the hydrophobic QVVAG hinge loop from the domain-swapping cystatin, stefin B, individually into the β-turn-β motifs of the single chain variant of the sweet protein monellin^40^ (MNEI). MNEI is similar structurally to stefin B (Fig. 1c) and other cystatin proteins^41^, but does not undergo domain swapping. We show that introducing the QVVAG motif into three different surface loops of MNEI results in domain-swapped dimerization, generating topologically-distinct domain-swapped dimers of MNEI. We then show that engineering the QVVAG stretch simultaneously into two different loops of MNEI creates two swappable domains in it, resulting in the formation of a double domain-swapped dimer. Thus, the QVVAG motif may be used to induce domain swapping in diverse β-hairpin containing proteins. Finally, we provide evidence for the generality of this design strategy by using the QVVAG motif to engineer domain swapping in three other proteins, which adopt folds that are distinct from the monellin/cystatin fold. Together, these results indicate that introduction of the QVVAG motif into surface β-loops of proteins is a simple mutational approach for the design of domain swapping in diverse β-hairpin proteins.

## Results

### Engineering domain swapping at loop1 of MNEI

**Fig. 2:**
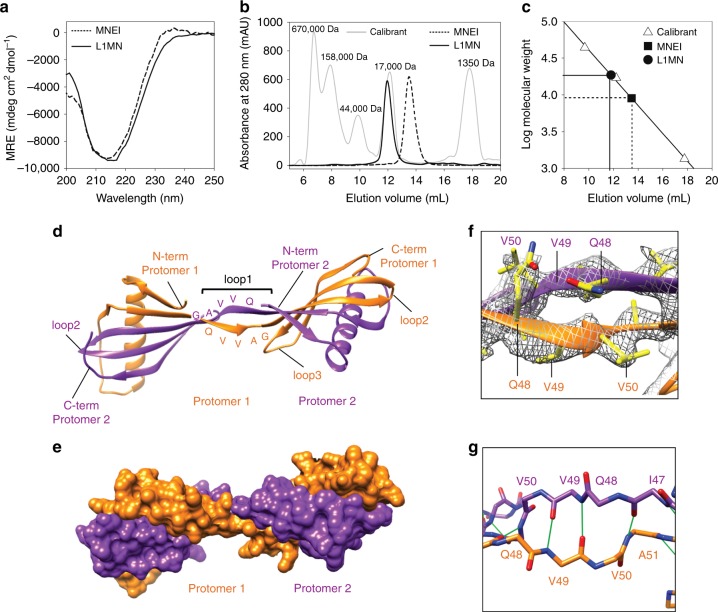
QVVAG segment from stefin B drives domain swapping in L1MN. **a** Far-UV CD spectra of wt MNEI and L1MN are shown. **b** Size exclusion profiles of wt MNEI and L1MN, at pH 7, are shown. The profile of the calibration mix (marker) is shown in gray. **c** Plot of the logarithm of molecular weight vs. elution volume generated using the elution profile of the calibration mix in (b) is shown. The data were fitted to a straight line (*r* = 0.99), and the apparent molecular weights of wt MNEI (■ ~10 kDa) and L1MN (● ~20 kDa) were determined. **d** Structure of the L1MN domain-swapped dimer is shown. Two polypeptide chains contributing to the swapped dimer (protomers) are shown in orange and purple. The N-termini and C-termini, and different loops of both the polypeptide chains contributing to the swapped dimer are indicated, except for loop3 of protomer 2, which is not clearly visible in the orientation depicted. **e** A space-filling model of the L1MN domain-swapped dimer is shown to highlight the quaternary arrangement of the protomers. **f** Zoomed in view of the hinge loop from **d**, showing the QVVAG stretch within the electron density map. The 2Fo-Fc simulated anneal composite omit map is shown at a contour level of 1.6σ. Side chain nitrogen atoms are colored blue, and oxygen atoms are colored red. All other side chain atoms are colored yellow. **g** The backbone trace of the hinge region is shown, and the backbone hydrogen bonds formed in the hinge region are shown by green lines. Source data are provided as a Source Data file

Although MNEI and stefin B are structurally similar (both fold to a β1-α1-β2-β3-β4-β5 topology, Fig. 1c), the functions of the two proteins are unrelated, and monellin is only 20% identical in sequence to stefin B. Additionally, stefin B domain-swaps^42^ but MNEI does not (Fig. 1c). The major conformational difference between the stefin B dimer and the MNEI monomer occurs in loop1, the loop connecting the β2 and β3 strands. While a longer loop1 forms a β-hairpin loop in MNEI, a shorter loop1 containing the QVVAG motif forms a hinge that connects the two swapped domains in the stefin B swapped dimer (Fig. 1b, c). A mutant variant of MNEI (L1MN) having a shorter loop1 carrying the stefin B QVVAG motif in loop1 (Fig. 1d) was designed, expressed and purified (see Methods and Supplementary Information). The circular dichroism (CD) spectrum of L1MN was similar to that of wild type (wt) MNEI (Fig. 2a), indicating that the secondary structure of monellin is conserved in L1MN. L1MN was run on a size exclusion chromatography (SEC) column, and was found to be dimeric (Fig. 2b, c). Neither monomer, nor higher order oligomers, were observed over a wide range of protein concentrations (10–750 μM). The molar mass of L1MN, determined from static light scattering experiments, was in excellent agreement with the calculated mass of the dimer (23 kDa and 21 kDa, respectively; Supplementary Table 1). These experiments indicate that L1MN exists almost completely as a dimer under the experimental conditions.

L1MN was crystallized and the crystals diffracted to a resolution of 2.5 Å (Supplementary Table 2). The crystal structure revealed that L1MN forms a symmetrical domain-swapped dimer. The two “subunits” of the dimer exchange “sub-domains” β1-α1-loopA-β2 and β3-loop2-β4-loop3-β5 (Fig. 2d-f, Supplementary Figure 1). Individually, both the subunits retain the fold of monellin, and superimpose with wt MNEI with a root mean square deviation (rmsd) of 1.2 Å (Supplementary Figure 2a). However, the conformation of loop1 is different in L1MN and wt MNEI. In contrast to the β-hairpin conformation observed in MNEI, loop1 exists as an extended β-strand in L1MN (Fig. 2d, f; Supplementary Figure 2a). Modeling a turn into the calculated electron densities for loop1 in L1MN, obtained from a composite omit map calculated by simulated-annealing, resulted in a poor fit and multiple steric clashes.

In the L1MN dimer, each monomeric “subunit” is composed of two polypeptide chains. Several van der Waals and hydrophobic contacts are formed between the QVVAG segments of the two polypeptide chains in the crossover region (Supplementary Table 3, Supplementary Figure 3). Moreover, symmetrical hydrogen bonds between the two QVVAG segments result in the formation of a new anti-parallel β-sheet between the two polypeptide chains, which further stabilizes the hinge region (Fig. 2g, Supplementary Table 3). Thus, a significant secondary interface (Fig. 1a) is present in the L1MN swapped dimer. Intriguingly, although both L1MN and stefin B dimerize by swapping subunits at the same interface, the angle between the subunits is significantly different in the two proteins (Supplementary Figure 2b). While the stefin B subunits are close in space, the L1MN subunits have swung away from each other.

### Engineering domain swapping at loop2 and loop3 of MNEI

**Fig. 3:**
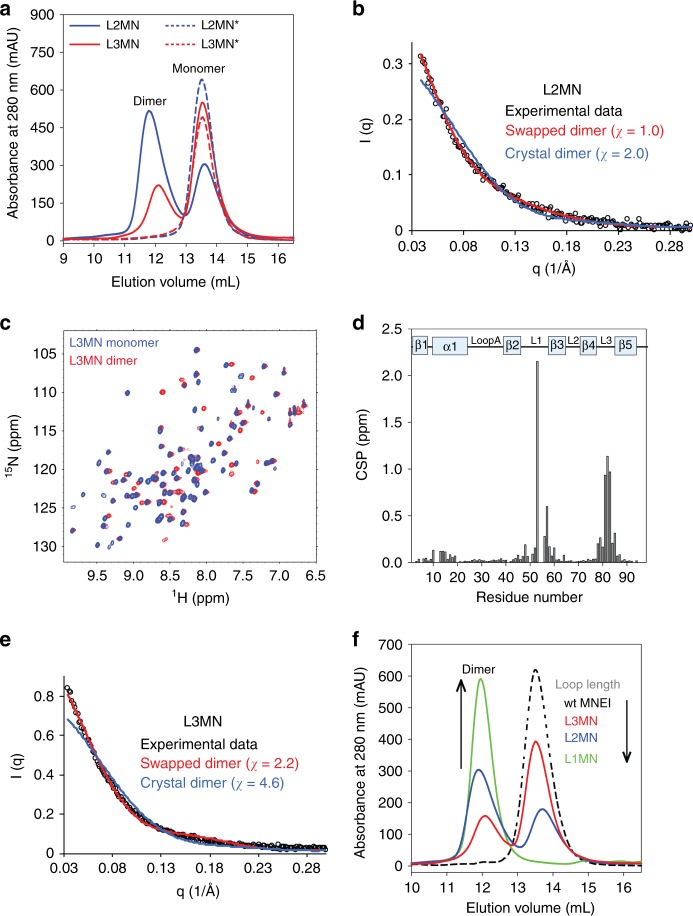
Domain swapping in L2MN and L3MN. **a** Size exclusion profiles of L2MN, L2MN*, L3MN, and L3MN* at pH 7, are shown. L2MN* and L3MN* are variants of L2MN and L3MN respectively, in which the QVVAG sequence was mutated to QVNAG. Elution volumes corresponding to the dimeric and monomeric species are indicated. **b** The experimental SAXS data for the L2MN dimer is shown against the calculated scattering curves for the L2MN domain-swapped dimer based on a structural model derived from symmetrized-SBM simulations of L2MN, and for the wt MNEI crystal-contact dimer (PDB ID: 1IV7). **c** ^15^N-HSQC spectra of L3MN monomer (blue) and dimer (red) align well for most resonances, indicating that their overall folds are similar. **d** Chemical shift perturbation (CSP) between the L3MN monomer and dimer is shown for each residue. A weighted chemical shift difference (^1^H and ^15^N) is plotted. For reference, the secondary structural arrangement of MNEI is indicated on the top. **e** The experimental SAXS data for the L3MN dimer is shown against calculated scattering curves for the L3MN domain-swapped dimer based on simulated models, and for the wt MNEI crystal-contact dimer (PDB ID: 1IV7). **f** Size exclusion profiles of wt MNEI, L1MN, L2MN, and L3MN are shown. Elution volumes corresponding to the monomeric and dimeric species are indicated. The fraction of the total protein that exists as a domain swapped dimer increases as the length of the target loop decreases (see Discussion). The total area under the elution peaks for each variant is comparable to the area under the elution peak for wt MNEI. Source data are provided as a Source Data file

Mutant variants of MNEI were designed with the QVVAG segment engineered into loop2 and loop3 (L2MN and L3MN, respectively; Fig. 1d). If indeed QVVAG can induce domain swapping, L2MN and L3MN should form topologically distinct domain-swapped dimers. Domain swapping in L2MN is expected to exchange subdomains β1-α1-loopA-β2-loop1-β3 and β4-loop3-β5. Domain swapping in L3MN is expected to produce a swapped dimer with the C-terminal β strands (β5) exchanged between the two polypeptide chains. Both the mutant variants were expressed and purified. SEC profiles of both L2MN and L3MN showed two well-separated peaks, corresponding to the hydrodynamic volumes of the monomer and the dimer (Fig. 3a). The dimer to monomer ratio was roughly 60:40 for L2MN, and 30:70 for L3MN. Static light scattering experiments on the dimeric fractions collected from SEC confirmed that L2MN and L3MN formed dimers (Supplementary Table 1). The CD spectra of the dimer and the monomer collected from the SEC for each mutant variant overlapped well (Supplementary Figure 4a, b), and were similar to the CD spectrum of wt MNEI, indicating that the secondary structure of MNEI is conserved in L2MN and L3MN.

A crystal contact-mediated dimer was observed in the wt MNEI crystal structure (PDB ID: 1IV7), in which the β-sheet forms the dimer interface. It was important to determine if the dimers observed in this study were formed by domain swapping or through the β-sheet interface. Despite a variety of conditions being tested, both L2MN and L3MN dimers did not yield good-quality crystals for diffraction. Moreover, L2MN dimers precipitated at high sample concentration, and could not be studied by nuclear magnetic resonance (NMR) spectroscopy. It should be noted that even though it was difficult to concentrate the L2MN dimer for NMR studies, the L2MN monomer to dimer ratio was unchanged over a concentration range of 10–200 μM.

The nature of the amino acid residues in the hinge region of a domain-swapping protein can control the degree of swapping^12,21^. A Val to Asn mutation has been shown to disfavor domain swapping in human cystatin C (hCC)^43^. Asn is a polar residue that is preferred in β-turns and substitution of the solvent-exposed hydrophobic Val in the “QIVAG” motif of hCC by Asn stabilized its monomeric conformation. We recently observed a similar effect in MNEI with a shortened loop1^34^. In an analogous manner, the monomeric conformation of L2MN could be stabilized by mutating QVVAG to QVNAG in loop2 (Fig. 3a), suggesting that the L2MN dimer is a domain-swapped dimer, where loop2 forms the hinge loop. As a control, the QVVAG to QVNAG mutation in loop1 of L1MN stabilized the monomeric conformation (Supplementary Figure 5). Finally, SAXS experiments were carried out on the L2MN dimers. The SAXS profile of the L2MN dimer closely fit the profile expected from a model with domain swapping at loop2 (*χ* = 1.0, Fig. 3b) (see Supplementary Methods), but not to the crystal contact dimer (*χ* = 2.0, Fig. 3b), providing further evidence that the L2MN dimers are formed by domain swapping.

The structure of the L3MN monomer was solved by X-ray crystallography and was found to be similar to the wt MNEI structure apart from minor differences in the fold of loop3 (Supplementary Figure 6). Since the L3MN dimer did not yield good-quality crystals, dimeric and monomeric L3MN were studied separately by NMR spectroscopy. The ^15^N-edited heteronuclear single-quantum coherence (HSQC) spectra of the monomer and dimer overlapped well (Fig. 3c), confirming that the overall fold of the monomeric and dimeric forms of L3MN is similar. The structural differences between the two were indicated by a few cross-peaks that did not overlap (Fig. 3c). The backbone chemical shifts of the monomer (Supplementary Figure 7) and dimer (Supplementary Figure 8) were assigned by standard triple resonance NMR experiments. The dimer peaks in the ^15^N-HSQC spectra were broader in comparison with those seen for the monomer (Supplementary Figure 9a), consistent with the larger size of the dimer. A comparison of the backbone amide chemical shifts revealed that the major chemical shift perturbations (CSPs) occur exclusively in loop1 and loop3 (Fig. 3d). While the loop3 CSPs are due to direct structural changes, the loop1 perturbations are probably indirect effects due to the spatial proximity of loop1 to loop3 (Fig. 1c). This is supported by a complementary observation that major CSPs between the monomeric and dimeric conformations of the domain-swapping cystatins, stefin A^13^ and hCC^44^, occur in both loop1 and loop3, even though domain swapping is known to occur only at loop1 in these proteins. Loop3 CSPs in stefin A and hCC were similarly suggested to be a result of the spatial proximity of loop3 to loop1. The predicted torsion angles (and secondary structure) of the L3MN dimer were similar to those of the L3MN monomer, except in loop3 (Supplementary Figure 9b, c). The predicted torsion angles indicated that loop3 adopted a β-strand conformation in the L3MN dimer, in contrast to being a β-turn in the L3MN monomer (Supplementary Figure 10a). Analysis of the medium-range NOEs confirmed the same (Supplementary Figure 10b). Since a mixed sample of labeled/unlabeled dimer could not be prepared, intermolecular NOEs could not be measured to solve the dimer structure. However, the SAXS profile of the L3MN dimer fit better to the loop3 domain-swapped dimer model structure (*χ* = 2.2, Fig. 3e) (see Supplementary Methods), than to the dimer observed in the crystal structure (*χ* = 4.6). Finally, similar to L2MN, the QVVAG to QVNAG mutation in loop3 of L3MN (L3MN*) led to the disappearance of the observed dimer (Fig. 3a). Altogether, the SEC, MALS, NMR and SAXS data indicate that L3MN forms a dimer by domain swapping, wherein loop3 acts as the hinge loop.

### Designing a double domain-swapped variant of MNEI

**Fig. 4:**
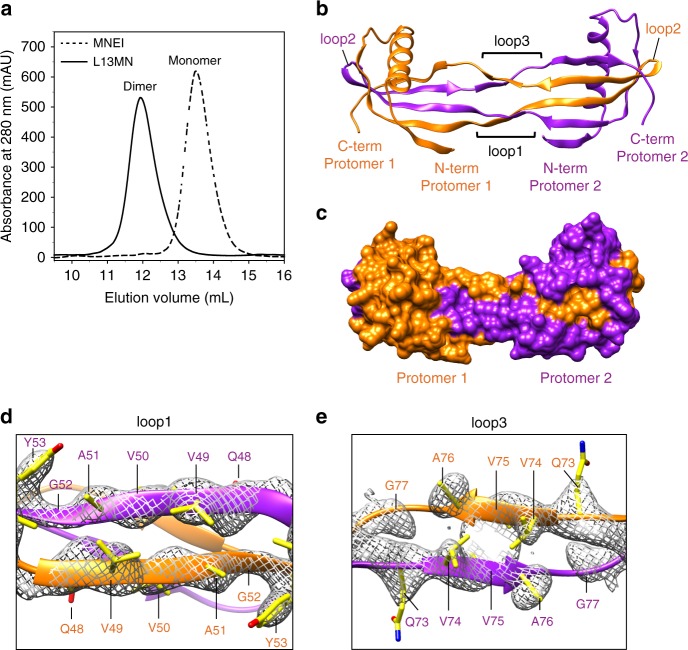
Crystal structure of L13MN. **a** Size exclusion profiles of L13MN and wt MNEI at pH 7 are shown. **b** Ribbon representation of the crystal structure of L13MN is shown. The two polypeptide chains contributing to the swapped dimer are colored in orange and purple. The three loops, and the N-termini and C-termini of each polypeptide chain are indicated. **c** A space-filling model of the L13MN domain-swapped dimer is shown to highlight the quaternary arrangement of the protomers. Zoomed views of the hinge loops along with electron density in loop1 and loop3 are shown in **d** and **e**, respectively. Side chain nitrogen atoms are colored blue, oxygen atoms are colored red, and the rest of the side chain atoms are colored yellow. The 2Fo-Fc simulated anneal composite omit maps are shown at a contour level of 1.6σ. Source data are provided as a Source Data file

The QVVAG segment was next introduced simultaneously into two different loops of MNEI, with the aim of designing two swappable domains in it. A variant of MNEI containing QVVAG segments in the two adjacent loops, loop1 and loop3, was designed (L13MN, Fig. 1d). L13MN was expressed, purified, and appeared folded from CD measurements (Supplementary Figure 4c). The SEC profile suggested that L13MN formed dimers (Fig. 4a), whose mass was confirmed by static light scattering experiments (Supplementary Table 1). L13MN was crystallized, and its structure was determined by X-ray crystallography to a resolution of 2.3 Å (Supplementary Table 2). The atomic structure of L13MN revealed a domain-swapped dimer, which was constructed by a novel criss-crossed arrangement of the two polypeptide chains contributing to the dimer (Fig. 4b, c). Both loop1 and loop3 acted as hinge loops, and adopted an extended conformation in the dimer (Fig. 4d, e and Supplementary Figure 11). Modeling a turn into the calculated electron densities for both loop1 and loop3 in L13MN resulted in a poor fit and multiple steric clashes. Swapping via loop1 results in an exchange of β1-α1-loopA-β2 and β3-loop2-β4-loop3-β5 sub-domains. Additional swapping via loop3 splits the β3-loop2-β4-loop3-β5 sub-domain further into β3-loop2-β4 and β5 sub-domains. This results in a reciprocal exchange of more than one sub-domain between the two L13MN polypeptide chains, forming a dimer where the β3-loop2-β4 hairpin from one polypeptide chain appears to have “inserted” into the neighboring polypeptide chain. Apart from multiple contacts within the QVVAG segments of loop1 and loop3, several contacts between these loops stabilize the swapped dimer (Supplementary Figure 12, Supplementary Table 4). Furthermore, ten hydrogen bonds were seen to have formed within the QVVAG segments at loop1 and loop3 (Supplementary Table 5). Thus, the introduction of the QVVAG motif in two adjacent β-hairpin loops in wt MNEI generated a reciprocal double domain-swapped dimer with multiple swapped interfaces.

The QVVAG stretch was next introduced into β-turn-β motifs of proteins unrelated to cystatins, to directly demonstrate that the QVVAG stretch can drive domain swapping in proteins that do not fold to the monellin/cystatin fold.

### Engineering domain swapping in MK-Ctd

**Fig. 5:**
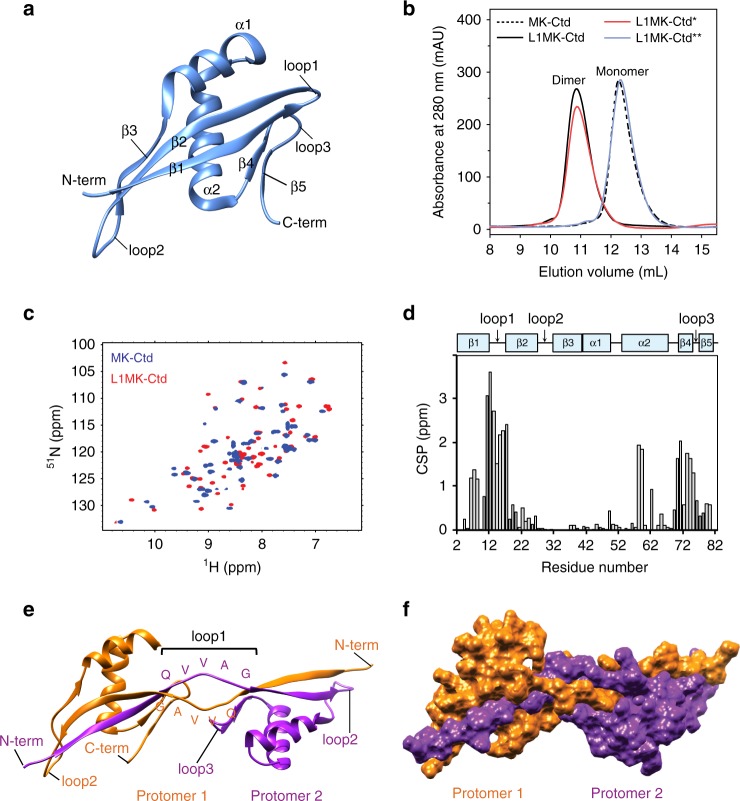
Domain swapping in MK-Ctd. **a** Structure of the C-terminal domain of the hyperthermophilic protein MK0293 (MK-Ctd) (PDB ID: 3C19) is shown. Different secondary structural elements, the N-termini and C-termini, and loop1, loop2, and loop3 of MK-Ctd are indicated. **b** Size exclusion profiles of MK-Ctd, L1MK-Ctd, L1MK-Ctd*, and L1MK-Ctd**, at pH 8, are shown. L1MK-Ctd* and L1MK-Ctd** are variants of L1MK-Ctd, in which the QVVAG sequence was mutated to QVNAG and QNNAG, respectively. Elution volumes corresponding to the monomeric and dimeric species are indicated. **c** An overlay of the ^15^N-HSQC spectra of the monomeric wt MK-Ctd (blue) and the dimeric L1MK-Ctd (red) is shown. **d** CSP between MK-Ctd and L1MK-Ctd is shown for each residue. A weighted chemical shift difference (^1^H and ^15^N) is plotted. For reference, the secondary structural arrangement of MK-Ctd is indicated on the top. **e** Ribbon representation of the solution dimer of L1MK-Ctd is shown. The two polypeptide chains contributing to the swapped dimer are colored in orange and purple. **f** A space-filling model of the L1MK-Ctd domain-swapped dimer is shown to highlight the quaternary arrangement of the protomers. Source data are provided as a Source Data file

The QVVAG stretch was next introduced into a β-hairpin loop in the C-terminal domain of the hyperthermophilic protein MK0293 (MK-Ctd), a protein domain derived from the protein MK0293 from *Methanopyrus kandleri* AV19 (PDB ID: 3C19, residues 98–178). MK-Ctd was chosen because it is unrelated to the cystatins, folds to a different topology^45^ (β1-β2-β3-α1-α2-β4-β5, Fig. 5a), and its purification protocol, biochemical properties and folding have been characterized in detail in our laboratories. The QVVAG stretch was engineered into loop1 of MK-Ctd, which is a tight β-turn (3 residues long) connecting two moderately extended β-strands, β1 and β2 (Fig. 5a). This mutant variant (L1MK-Ctd) was expressed and purified, and it appeared well-folded from CD measurements (Supplementary Figure 13). The SEC profile suggested that L1MK-Ctd was entirely dimeric in solution (Fig. 5b), which was confirmed by static light scattering experiments (Supplementary Table 1).

The L1MK-Ctd dimer was analyzed by NMR spectroscopy. The ^15^N-edited HSQC spectrum of the L1MK-Ctd dimer showed well-dispersed peaks (Fig. 5c), where several resonance cross-peaks were shifted from those of wt MK-Ctd (Fig. 5c). The backbone resonances were assigned for both wt MK-Ctd and L1MK-Ctd by standard triple resonance NMR experiments (Supplementary Figure 14 and 15). Only a single set of resonances was observed in the L1MK-Ctd spectra, indicating that L1MK-Ctd is a symmetric dimer. A comparison of the backbone amide chemical shifts of the monomeric wt MK-Ctd and dimeric L1MK-Ctd revealed that the major chemical shift perturbations between the two proteins were localized to three distinct positions (β1-loop1, a few residues within α2, and β4-loop3-β5, Fig. 5d). However, the predicted secondary structure (and torsion angles) of only loop1 residues differed between the two (Supplementary Figure 16); loop1 adopted a β-strand conformation in the L1MK-Ctd dimer, but a β-turn conformation in the monomeric wt MK-Ctd (Supplementary Figure 16). CSPs at other locations can be attributed to the physical proximity of β4-loop3-β5 to loop1, and docking of α2 against the β1–β2 hairpin (Fig. 5a). Therefore, similar to L3MN, NMR data along with SEC and light scattering data, suggests that L1MK-Ctd forms a dimer by domain swapping, wherein loop1 acts as the hinge loop.

Finally, the solution structure of L1MK-Ctd was determined by NMR. ^13^C-edited and ^15^N-edited NOESY-HSQC experiments were carried out on uniformly ^13^C, ^15^N-labeled L1MK-Ctd. The NOESY cross-peaks in the spectra provided both the intra-protomer connectivities and the inter-protomer connectivities. Exclusive NOE cross-peaks resulting from inter-protomer connectivities were extracted from filtered NOESY experiments. Briefly, heterolabeled dimeric L1MK-Ctd was prepared by refolding a 1:1 mixture of unfolded uniformly ^15^N/^13^C-labeled and unlabeled L1MK-Ctd proteins (see Supplementary Methods), which is therefore expected to be a mixture of 25% labeled-labeled, 50% labeled-unlabeled, and 25% unlabeled–unlabeled dimers. The SEC profile of the refolded heterolabeled sample showed that >90% of the total protein existed as a dimer (Supplementary Figure 17). 92 inter-protomer connectivities could be assigned from the various NOESY experiments. About 102 dihedral angle restraints (φ and ψ) were determined per protomer using the ^1^H_α_, ^15^N, ^13^C_α_, ^13^C_β_, and ^13^CO chemical shifts and the program TALOS+^46^. Twenty-eight intra-protomer helix hydrogen bonds were inferred per protomer from the standard secondary structure of the protein based on NOE patterns. No hydrogen bond restraints were used for β-strands to avoid bias for swapping. Using the NOE based distance restraints, dihedrals and hydrogen bond restraints, the structure of L1MK-Ctd dimer was calculated in Xplor-NIH^47^. Supplementary Figure 18a shows the twenty lowest energy structures, and Fig. 5e shows the lowest energy structure. The structural statistics are provided in Supplementary Table 6.

The solution structure of L1MK-Ctd dimer revealed a domain-swapped dimer, formed by the exchange of the N-terminal β1 strand between the two polypeptide chains (Fig. 5e, f), in which loop1 is extended into a β-strand conformation, as opposed to a turn in the wt monomeric protein. The individual subunits in the dimer superimpose well with monomeric wt MK-Ctd (Supplementary Figure 18b). The rmsd of twenty lowest energy structures within each protomer is 1.1 Å, whereas the rmsd of the complete dimer is 2.1 Å (Supplementary Table 6), suggesting that the hinge region between the two protomers is conformationally flexible. When the structures were aligned for one subunit, the other subunit spans an angle of ~24°, which indicates the range of conformational dynamics between the subunits (Supplementary Figure 18c). Several van der Waals and hydrophobic contacts are formed between the QVVAG segments of the two polypeptide chains in the crossover region (Supplementary Figure 18d and Supplementary Table 7). Unlike L1MN and L13MN, the two QVVAG segments at the hinge do not form a complete anti-parallel β-strand. This is probably because the hinge buries the first valine side chain of the QVVAG motif to shield the hydrophobic side chain (Supplementary Figure 18d, e).

Interestingly, the QVVAG to QVNAG mutation in L1MK-Ctd did not result in stabilization of its monomeric conformation (Fig. 5b); similar to L1MK-Ctd, the QVNAG variant, L1MK-Ctd*, was exclusively dimeric in solution. A closer look at the orientation of the side chain groups of the QVVAG residues in the new anti-parallel β-sheet formed between the two polypeptide chains in the crossover region revealed that the side chain group of the central valine was solvent exposed (Supplementary Figure 18d), which might explain why the V → N mutation at this position did not affect the oligomeric status of L1MK-Ctd. However, the hydrophobic side chain group of the preceding valine is buried in the secondary interface (Supplementary Figure 18d, e), and the mutation of the QVVAG motif to QNNAG resulted in complete reversal of the observed domain-swapped dimerization in L1MK-Ctd (Fig. 5b). A similar orientation of the side chains of the two Val residues is seen at the secondary interface in L1MN (Fig. 2f, g, Supplementary Figure 3); the hydrophobic side chain of the central Val residue is solvent accessible, while that of the first Val is buried at the interface. The V to N mutation at the first Val is therefore expected to diminish dimerization more effectively due to disruption of the hydrophobic interactions. Therefore, we made a similar QVVAG to QNNAG mutation in L1MN, and the resulting variant (L1MN**) was also found to be completely monomeric (Supplementary Figure 5).

### Engineering domain swapping in Sso7dv and UBQ

The QVVAG motif was then introduced into a β-hairpin motif of Sso7d (a small DNA binding protein^48,49^) and human ubiquitin (UBQ). These proteins were chosen because they adopt folds that are structurally unrelated to the monellin/cystatin fold, and their purification protocols, biochemical properties, and structural details have already been characterized in our laboratories.

**Fig. 6:**
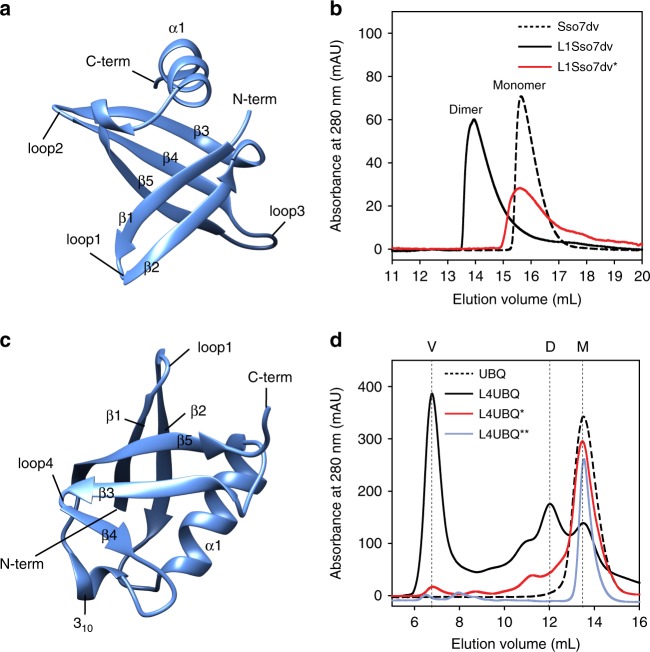
Domain swapping in Sso7dv and UBQ. **a** Structure of Sso7d (PDB ID: 1BNZ) is shown. Different secondary structural elements, the N-termini and C-termini, and loop1, loop2, and loop3 of Sso7d are indicated. **b** Size exclusion profiles of Sso7dv, L1Sso7dv, and L1Sso7dv*, at pH 7.5, are shown. L1Sso7dv* is a variant of L1Sso7dv, in which the QVVAG sequence was mutated to QVNAG. Elution volumes corresponding to the monomeric and dimeric species are indicated. **c** Structure of human ubiquitin (UBQ) (PDB ID: 1UBQ) is shown. Different secondary structural elements, the N-termini and C-termini, and loop1 and loop4 of UBQ are indicated. **d** Size exclusion profiles of UBQ, L4UBQ, L4UBQ* and L4UBQ** at pH 8.3, are shown. L4UBQ* and L4UBQ** are variants of L4UBQ, in which the QVVAG sequence was mutated to QVNAG and QNNAG, respectively. The elution volumes of the monomer (M), dimer (D) and the void fraction (V) are indicated. Source data are provided as a Source Data file

Sso7d (SCOPe ID: 54163) has an SH3-like fold^50^, and folds to a β1-β2-β3-β4-β5-α1 topology (Fig. 6a). We used a variant of Sso7d, Sso7dv, whose structure was recently determined in our laboratories. Sso7dv is derived from a Sso7d scaffold^51^ where the residues on the DNA binding interface have been randomized, thereby abolishing the DNA-binding ability of the scaffold (see Supplementary Methods for the scaffold sequence). The QVVAG motif was introduced into the loop connecting β1–β2 (loop1) of Sso7dv (Fig. 6a). This mutant (L1Sso7dv) was expressed and purified (see Supplementary Methods). L1Sso7dv appeared folded from CD measurements (Supplementary Figure 19). The SEC profile of L1Sso7dv showed that the protein was predominantly dimeric (Fig. 6b). To confirm that the observed dimerization was a result of domain swapping, the oligomeric status of L1Sso7dv* (QVVAG to QVNAG mutation in L1Sso7dv) was assessed. Figure 6b shows that L1Sso7dv* was entirely monomeric, which indicates that L1Sso7dv dimers are likely domain-swapped dimers.

UBQ adopts a β-grasp fold, and folds to a β1-β2-α1-β3-β4-3_10_-β5 topology (Fig. 6c). The QVVAG stretch was engineered individually into the loops connecting β1-β2 (loop1) or β3-β4 (loop4) in UBQ (Fig. 6c). The loop1 variant of UBQ (L1UBQ) was entirely monomeric in solution (Supplementary Figure 20a). Unfolding and refolding of purified monomeric L1UBQ, but not wt UBQ, at high protein concentrations (~ 1 mM; see Supplementary Methods) led to the conversion of <5% of the protein into a dimer (Supplementary Figure 20a), whose CD spectrum overlapped well with that of wt UBQ (Supplementary Figure 20b), indicating that the dimer possibly forms by domain swapping. However, because the proportion of the observed dimer was insignificant, L1UBQ was not characterized further. In contrast to L1UBQ, the loop4 variant of UBQ (L4UBQ) was isolated from the insoluble fraction of the cell lysate under denaturing conditions followed by refolding (see Supplementary Methods). The SEC profile of the purified L4UBQ showed multiple peaks (Fig. 6d), indicating that the protein exists as a mixture of different oligomeric forms. Static light scattering experiments on the oligomeric fractions collected from SEC confirmed that L4UBQ formed dimers and higher-order multimers (Supplementary Table 1). Further, ensemble measurements like circular dichroism indicated that the secondary structure of UBQ is conserved in these multimers (Supplementary Figure 21a). The oligomeric fraction eluting in the void volume (Fig. 6d) was analyzed by cryo-electron microscopy (Supplementary Figure 21b), which revealed that the oligomeric fraction is a heterogeneous mixture of linear multimers of varying lengths. The absence of thioflavin T (ThT) binding to the L4UBQ oligomeric species ruled out the possibility of an ordered amyloid-like (cross-β sheet) structural arrangement^52^ in these oligomers (Supplementary Figure 21c). Finally, the QVVAG to QVNAG mutation was found to significantly stabilize the monomeric conformation of L4UBQ (Fig. 6d). The QVVAG to QNNAG mutation in L4UBQ resulted in complete monomerization (Fig. 6d), similar to our observations with L1MN and L1MK-Ctd. These results suggest that multimerization of UBQ, observed upon introduction of the QVVAG motif into its loop4, appears to be a result of (open-ended) domain swapping.

## Discussion

Here, we have shown that engineering the QVVAG sequence from stefin B into any of the three β-turn-β loops of MNEI induces dimerization through domain swapping. The QVVAG (or QXVXG) motif is not found naturally in structural contexts similar to those in which it was engineered into the variants L2MN and L3MN (i.e., in the loop connecting β3–β4 or β4–β5). The induction of domain-swapped dimerization in L2MN and L3MN suggests that the QVVAG motif can drive domain swapping in diverse β-hairpin containing proteins, which may fold to topologies/folds different from the monellin/cystatin fold. Experiments with structurally and functionally unrelated proteins, MK-Ctd, Sso7dv, and UBQ, lend support to this claim because dimerization/oligomerization of these proteins was observed when the QVVAG motif was introduced into their β-turn-β loops as well. Thus, the QVVAG stretch appears to be a versatile motif for engineering domain-swapped oligomerization in proteins. However, given how intricately the phenomenon of domain swapping is linked to protein folding^7,12^, experiments on a bigger set of proteins will be required to establish the extent of generality of this method and to completely understand the factors modulating the efficiency of domain-swapping using the QVVAG sequence. Factors that could affect the efficiency of this approach include the length and solvent exposure of target loops, the global stability of the target protein, the presence of regions of structural weakness in the protein^53^, the mechanism of protein folding dictating where along the folding route the protein dimerizes, and the presence of partially unfolded intermediates which promote self-assembly. We discuss some of these factors next.

A comparison of the structures of L1MN and several structurally homologous (swapped at loop1) domain-swapped cystatins^35,39,42,54^ shows that the configuration of the two QXVXG motifs which enable dimerization are variable across the structures with both stacked (e.g., L1MN: C_α_ Q–Q distance of ~6 Å) and staggered (e.g., Stefin B, PDB ID 2OCT: C_α_ Q–Q distance of ~19 Å) arrangements being present. The angle between the two subunits of the domain-swapped structures is also variable. For example, L1MN has a wider angle (~160°) between its two subunits as compared to stefin B (~42°) (Supplementary Figure 2b). Further, the percent population of dimers (and the oligomerization behavior) in the several different loop variants of MNEI, MK-Ctd, Sso7dv and UBQ is variable. Together, these observations imply that domain swapping is dependent on the structural context of the QVVAG sequence.

Earlier studies had indicated that the reason behind QVVAG-induced domain swapping is the presence of hydrophobic residues (VVA) in a solvent-exposed loop. It has been hypothesized that the solvent exposure of the hydrophobic residues creates strain and does not allow the loop to fold back upon itself. Support for this hypothesis comes from the fact that the central V of the QVVAG motif in the monomeric conformation of Stefin B (Fig. 1c) is strained and lies in a disallowed region of the Ramachandran plot^35^. One way to stabilize such solvent-exposed loop hydrophobic residues is domain-swapped dimerization^35,36,54^. The stacked arrangement of the QVVAG stretches observed in the L1MN and L13MN crystal structures and L1MK-Ctd NMR structure reduces the solvent-exposure of these hydrophobic residues, but is also likely to contribute to stability due to the formation of a new anti-parallel β-sheet between the two polypeptide chains in the crossover region (secondary interface). However it is difficult to delineate the relative contribution of these two factors in driving domain swapping because Val is both hydrophobic and has a high propensity to form β-sheets due to its β-branched side chain.

Domain-swapping which occurs due to the hydrophobicity of the QVVAG motif and its surface exposure can be modulated in three ways. The first, the most direct approach, is through the reduction in hydrophobicity of the exposed residues. For example, a mutation of the apex V to the polar N in the solvent exposed QIVAG motif of human cystatin C (hCC)^43^ reduces the destabilization due to solvent exposure, shifts the N to a “generously allowed” region of the Ramachandran plot and stabilizes the monomer. A similar monomer stabilization is seen in the QVVAG to QVNAG or QVVAG to QNNAG mutants in our experiments (Figs. 3a, 5b, 6b, 6d, Supplementary Figure 5).

The second approach to monomer stabilization is through a reduction in the solvent-exposure of the hydrophobic QVVAG loop. The monomeric conformation of L1UBQ was not destabilized enough to induce domain swapping in it (Supplementary Figure 19a) possibly because the QVVAG stretch was introduced into a loop with low solvent-exposure due to the presence of a hydrophobic network around it. The central valine in the QVVAG stretch replaces Leu-8 of UBQ, which is a part of the functionally important ubiquitin surface hydrophobic patch comprised of Leu-8, Ile-44, and Val-70^55–57^.

The third method for monomer stabilization is a change in the length of the QVVAG containing hinge loop. Hinge loop length is known to manipulate the strain in proteins^6,7,11,25,58^. Longer loops disfavor domain swapping due to increased conformational plasticity and very few domain-swapping proteins are known to have hinge loops longer than 5–6 residues^59^. For MNEI, we observed an inverse correlation between the length of the loop into which the QVVAG motif was introduced (e.g., loop1 in L1MN (1 residue long) < loop2 in L2MN (2 residues long) < loop3 in L3MN (6 residues long); calculated using^60^) and the extent of observed dimerization in the resultant variant (dimer population: L1MN > L2MN > L3MN, Fig. 3f). Together, our results indicate that the insertion of the QVVAG stretch is most likely to succeed in promoting domain-swapping induced multimerization in proteins which have tight and solvent-exposed polar β-turns.

A high wt protein stability could overcome the effects of destabilization due to the solvent exposure of the QVVAG motif and also lead to monomer stabilization. For instance, the designed thermostable (*T*_m_ ~101 °C) peptide display scaffold Adhiron^61^ (PDB ID: 4N6T), derived from a photocystatin^62^ consensus sequence, is monomeric despite the presence of the QVVAG motif. Nevertheless, a correlation between protein stability and efficiency of dimerization was not observed for the four proteins studied here (stability of the four proteins was in the range 3–9 kcal mol^-1^, measured at 25 °C, pH 7, Supplementary Figure 22). It is also possible that the introduction of the QVVAG motif into tight β-turns alters the packing interactions between the β-strands of the target β-hairpin motif, selectively destabilizing the monomeric conformation thereby promoting domain swapping. We compared the backbone H-bond distances between the intramolecularly formed β-sheets, β2–β3 in wt MNEI and β1–β2 in wt MK-Ctd, with the H-bond distances in the intermolecularly formed β-sheets in the respective domain swapped dimers (L1MN and L1MK-Ctd), and found them to be similar (Supplementary Table 8). This indicates that domain-swapping in at least L1MN and L1MK-Ctd is not due to altered β-sheet pairing.

The versatility of the QVVAG motif is highlighted by the fact that loop engineering using this motif enabled the design of several different modes of domain swapping in the different monomeric proteins used in this study, including single domain swapping, double domain swapping and open-ended domain swapping. L13MN is the first instance of a designed double domain-swapped protein. The parallel arrangement of the two subunits in the L13MN dimer results in the stacking of four QVVAG stretches, generating a small hydrophobic patch at their interface. The stacked arrangement creates a significant secondary interface. Consequently, the structure of the L13MN swapped dimer is more integrated than the structure of the L1MN dimer. The number of contacts (including van der Waals contacts and hydrogen bonds) observed at the secondary interface in L1MN is 15 (Supplementary Table 3), whereas the same in L13MN is 66 (Supplementary Tables 4 and 5), indicating significantly higher integration in L13MN. Linking the two protomers of L13MN covalently via a flexible linker could create a single polypeptide that folds to a structure that is the same as that of the L13MN dimer. Thus, domain swapping can be used to design novel folds with minimal computational intervention by reshaping existing folds and exploiting evolutionarily optimized interactions and interfaces.

The induction of multimerization by engineered open-ended domain swapping has been achieved earlier for a few proteins^11,23,31,63^, however, the rational design of open-ended domain swapping is more difficult as compared to the design of reciprocal domain swapping in proteins. We next discuss the potential reasons for the observation of multimerization in L4UBQ and not the other mutants. It is possible that the structural malleability in the region following loop4 (which includes the short β-strand, β4, and a short 3_10_ helix; Fig. 6c) in UBQ promotes multimerization due to the increased kinetic accessibility of such conformations^12^. Higher-order domain-swapped oligomers have been observed earlier upon introduction of greater flexibility in the hinge loop, by increasing the length or altering the amino acid composition, in a few proteins^11,31,32,63^. Further, a comparison of the structures of the dimeric N-terminal fragment of human UBQ^64^ (PDB ID: 1GJZ) and the domain-swapped dimer of a ubiquitin-like plant protein, ATG12^65^ (PDB ID: 1WZ3), where loop4 exists in an extended conformation, suggests that it is plausible that there are two distinct modes of domain swapping possible for L4UBQ, a phenomenon which could also increase the probability of generating higher-order oligomers. Structural characterization of the L4UBQ multimers should aid in further understanding the factors that contribute to the observed distinct domain-swapping behavior of L4UBQ and the rational design of higher-order oligomerization.

In summary, the design of dimerization (or oligomerization) through domain swapping is advantageous because it requires the mutation of only a few amino-acid residues^12,66^. The similarity of the swapped structure with the monomer is expected to reduce the inadvertent loss of protein function that can occur when many amino acid residues are mutated to design a new protein-protein interaction interface. The lack of a clear strategy for inducing domain swapping has hindered its use in the design of protein oligomerization and assembly. Here, we provide a possible strategy, the insertion of the QVVAG stretch into tight and solvent-exposed β-turns of proteins. The design of domain swapping using the QVVAG stretch does not require the use of computational tools, and given the minimal perturbation to a sequence that this strategy presents, it could be easily integrated with existing methods for the design of protein assemblies and to yield a rich complexity of protein nanostructures.

## Methods

### Design of the loop variants

Loop variants of MNEI, MK-Ctd, Sso7dv, and UBQ with the QVVAG motif engineered into different loops were constructed by site-directed mutagenesis (SDM), using a single mutagenic (forward) primer^67^. In L1MN, residues 51–56 (EGFREI) were deleted (MNEIΔ6^Asn^)^34^ followed by mutation of the flanking YENK stretch to QVVA (48–51) to convert residues number 48–52 to QVVAG. L1MN is 91 residues long. In L2MN, residues 66–70 were mutated from YASDK to QVVAG. In L3MN, residues 79–83 were mutated from DYKTR to QVVAG. Both L2MN and L3MN are 97 amino acid residues long. In L13MN, residues 73–77 (which correspond to residues 79–83 in wt MNEI) in L1MN were mutated from DYKTR to QVVAG. L13MN is 91 residues long. In L1MK-Ctd, residues 13–17 were mutated from YGERE to QVVAG. In L1Sso7dv, residues 7–12 were mutated from KYKGEE to QVVAG. In L1UBQ, residues 6–9 were mutated from KTLT to QVVA to convert residues number 6–10 to QVVAG. In L4UBQ, residues 44–48 were mutated from IFAGK to QVVAG. Primers for SDM were obtained from BioServe, India. The purification methods for MNEI, MK-Ctd, Sso7dv, and UBQ loop variants are described in detail in the Supplementary Methods.

### Buffers and reagents

All the chemicals used for the study were of high purity grade, and were procured from Sigma. Experiments with MNEI and its loop variants were carried out using 50 mM phosphate buffer, containing 250 μM EDTA and 1 mM DTT, at pH 7. Experiments with MK-Ctd and its loop variants were carried out using 20 mM Tris-HCl buffer, containing 500 mM GdnHCl, at pH 8. Experiments with Sso7dv and its loop variants were carried out using 50 mM phosphate buffer, containing 200 mM NaCl, at pH 7.5. Experiments with UBQ and its loop variants were carried out using 20 mM Tris-HCl buffer, containing 150 mM NaCl, at pH 8.3.

### Size exclusion chromatography

The oligomeric status of different proteins was determined using a Superdex 75 10/300 GL size exclusion column (which can resolve proteins in the molecular weight range 3–70 kDa) on an ÄKTA FPLC. The column was run at 0.5 ml min^-1^, and protein elution was monitored at 280 nm. The apparent molecular weight of different proteins was estimated from a calibration curve generated using a Bio-Rad gel filtration standard. For all the protein variants, a representative SEC profile is reported. Proteins were lyophilized immediately after purification and stored at −20 °C. These proteins were later dissolved in appropriate buffers and their oligomeric status was analyzed by SEC. Each variant was expressed and purified at least three times to verify the reproducibility of the observed trends.

### Circular dichroism measurements

The far-UV circular dichroism (CD) spectra of MNEI, MK-Ctd, Sso7dv, and UBQ, and their different loop variants, were acquired on a Jasco J-815 spectropolarimeter, using a protein concentration of 10–20 μM in a 1 mm path length quartz cuvette, with a bandwidth of 1 nm, a scan speed of 50 nm min^−1^, and a digital integration time of 1 s. Fifteen scans were averaged for each sample.

### Protein crystallization

2 μl of 2–4 mg ml^−1^ of protein (L1MN, L13MN, and L3MN monomer) was mixed with 2 μl of reservoir solution, and set for crystallization using the hanging drop vapor diffusion method at 4 °C. The reservoir solution contained 8–12% (wt/vol) PEG 8000 and 50 mM sodium phosphate at pH 6.4–6.8. Crystals of L1MN dimer and L3MN monomer grew to their maximum size in 4–5 days. Crystals for L13MN appeared only after a week. Crystals were cryoprotected by soaking them in a solution containing 15% (wt/vol) PEG 8000 and 50 mM sodium phosphate pH 6.4–6.8, supplemented with glycerol that was increased in steps of 5% from 0 to 30% (vol/vol). At each incremental step, crystals were dehydrated at 4 °C for 6–12 h. Crystals were then flash frozen in liquid N_2_.

### X-ray diffraction data collection and structure determination

Data collection for L1MN crystals was carried out at 100 K at the Proxima-1 beamline of the Soleil Synchrotron France, on a PILATUS 6 M detector using a beam of wavelength 0.97857 Å^68^. Data collection for L13MN crystals was carried out at 100 K at the ID30A-1/MASSIF-1 beamline in ESRF Synchrotron France, on a PILATUS3 2 M detector using a beam of wavelength 0.966 Å^69^. Data collection for L3MN monomer was carried out on a Rigaku FR-X machine (1.5418 Å wavelength) at 100 K. Data were indexed and integrated using the XDS software^70^. Scaling and merging of diffraction intensities were carried out using the POINTLESS and AIMLESS software in the CCP4 package^71^. The structures of the different MNEI variants were solved by molecular replacement using wt MNEI (PDB ID: 1IV7) as a search model, and the program MOLREP^72^. The model was refined iteratively using the program PHENIX^73^, and manually rebuilt using the program COOT^74^. Water molecules were modeled using COOT. The model was partitioned into multiple groups, identified from the TLSMD server^75^, and was subjected to TLS (Translation/Libration/Screw) vibrational motion refinement in PHENIX. Simulated Annealing Composite Omit 2Fo-Fc electron density map was generated using PHENIX. Cartesian dynamics with starting temperature of 5000 K was used for simulated annealing. The map was calculated over the entire unit cell and about 5% of the structure was omitted at a time. The map was then used to manually rebuild and correct the structure model of any discrepancy. The Fo-Fc map was generated similarly. All the structures had good geometries, with 93–95% residues in the favored region and 0–0.5% residues in the outlier regions of the Ramachandran Plot. Clashscores, calculated by Molprobity^76^, were <6 for all structures. Diffraction data and refinement statistics are summarized in Supplementary Table 2.

### NMR experiments and structure determination

The monomeric and dimeric fractions of ^13^C, ^15^N-labeled L3MN were separated using size exclusion chromatography. Each fraction was concentrated to ~400 μM in 20 mM phosphate buffer at pH 7.2, containing 0.03% sodium azide. For the backbone assignment of the L3MN dimer, NMR spectra were recorded at 298 K on an 800 MHz Bruker Avance III HD spectrometer, equipped with a cryo-probe head. All NMR samples contained 10% D_2_O (vol/vol). Standard HN(CO)CACB, HNCACB, HNCO, HNCA and HN(CO)CA NMR triple resonance 3D experiments were used for backbone assignments. All NMR data were processed using NMRPipe^77^, and analyzed by the Sparky software^78^. Following peak picking of the backbone experimental data in Sparky, the data were assigned by the PINE NMR-server^79^ and then verified, corrected and completed manually. The backbone assignments are 97% complete. The TALOS+ software^46^ was used to predict the ϕ, ψ torsion angles from the assigned ^1^H_α_, ^15^N, ^13^C_α_, ^13^C_β_, and ^13^CO chemical shifts, for both the monomer and the dimer. The backbone resonance assignment of ^13^C, ^15^N-labeled monomeric wt MK-Ctd, and the ^13^C, ^15^N-labeled L1MK-Ctd dimer were carried out by standard triple resonance 3D experiments, as mentioned above. The torsion angles and secondary structure were calculated as described above. The side chain resonances of wt MK-Ctd were assigned by triple resonance ^13^C-edited HSQC, (H)CC(CO)NH and H(CCCO)NH experiments. Some side chain resonances of L1MK-Ctd dimer were assigned by comparing peaks between ^13^C-edited HSQC of the mutant dimer and the wt MK-Ctd. The rest of the side chain resonances were assigned by using HAHB(CO)NH and HCCH-TOCSY experiments. ^13^C-edited and ^15^N-edited NOESY-HSQC experiments were carried out on uniformly ^13^C, ^15^N-labeled L1MK-Ctd. A ^12^C/^14^N,^13^C/^15^N heterolabeled dimeric sample was prepared by unfolding the unlabeled (^12^C/^14^N) and uniformly ^13^C/^15^N labeled dimeric proteins separately, by incubating them in 7 M GdnHCl (in 20 mM Tris-HCl, pH 8) for 3 h at a concentration of ~0.7 mM. Following this, equal volumes of the two proteins were mixed and left at room temperature for 15 min. Finally, refolding was initiated by reducing the denaturant concentration to ~0.5 M by 15-fold dilution in the refolding buffer (20 mM Tris-HCl, pH 8), which decreased the total protein concentration to ~50 μM. The mixture was incubated overnight for equilibration. The SEC profile of the refolded mixture prepared in this manner showed that >90% of the total protein existed as a dimer (Supplementary Figure 17), which was collected and analyzed by NMR spectroscopy. The ^15^N-edited HSQC and ^13^C-edited HSQC compared well between the standard sample and refolded heterolabeled sample, indicating that the refolding protocol did not disturb the fold of the protein. ^13^C/^15^N-F1-filtered, ^13^C-F3-edited-NOESY-HSQC and ^13^C/^15^N-F1-filtered, ^15^N-F3-edited-NOESY-HSQC data were collected with the heterolabeled sample to obtain intermolecular restraints between the two protomers. About 800 NOE based distance restraints and 102 dihedral restraints were obtained per protomer. Given L1MK-Ctd is a symmetric dimer with an exclusive set of peaks in the HSQC, the NOE and dihedral restraints were applied to both the protomers during the structure calculation in Xplor-NIH^47^. At the refinement stage, 28 helix hydrogen bonds were added based on the NOE patterns. Two hundred structures were calculated in Xplor-NIH^47^ by simulated annealing. The twenty lowest energy structures have been deposited in the PDB server (PDB ID: 6IWJ).

### SAXS data collection and analysis

SAXS measurements were carried out using a BIOSAXS-1000 small-angle X-ray scattering Kratky camera system, installed on a Rigaku microfocus X-ray generator (1.5418 Å wavelength). Purified L2MN and L3MN dimer fractions at 2–3 different concentrations were subjected to X-rays for 30 min each at 25 °C. Buffer scattering, recorded under identical conditions, was subtracted from the scattering of the protein sample. The scattering curve was fitted to structural models using the software FoXS^80^. Data were analyzed using the Primus and Gnom software in the ATSAS suite (EMBL Hamburg). The radius of gyration (*R*_g_) was averaged for all concentrations to obtain the mean value. The *R*_g_ values were found to be 26.5 ± 2.2 Å and 27.4 ± 2.8 Å (*n* = 3, mean ± s.e.m.) for the L2MN and L3MN dimers, respectively.

### Electron microscopy

Three microliter of the L4UBQ void fraction at 1 mg ml^−1^ was applied to a glow discharged Quantifoil 1.2/1.3 holey carbon grids. Grids were frozen in liquid ethane with a Vitrobot (FEI) at 100% humidity and 18 °C for 3 s. The grids were transferred to Krios Autogrids and images were acquired on a Titan Krios with a Falcon 3 detector at a nominal magnification of 47,000× (calibrated magnification—78651) resulting in 1.78 Å per pixel. The total exposure was 3 s, and dose was ~70 e^-^ per Å^2^.

### Reporting summary

Further information on experimental design is available in the Nature Research Reporting Summary linked to this article.

## Supplementary information

Supplementary Information

Reporting Summary

Source Data

## Data Availability

The coordinates and structure factors for L1MN, L13MN, and L3MN monomer have been deposited in the Protein Data Bank (PDB), under the accession codes 5YCU, 5YCW, and 5YCT, respectively. The coordinates of the NMR models of the L1MK-Ctd dimer have been deposited in the PDB, under the accession code 6IWJ. NMR data for the L3MN monomer, L3MN dimer and L1MK-Ctd dimer are deposited in the BMRB under the accession codes 27248, and 27247 and 36222, respectively. The source data underlying Figs. 2a–c, 3a, b, d–f, 4a, 5b, d, 6b, d, and Supplementary Figures 4, 5, 9, 13, 16, 17, 19, 20, 21a, 21c and 22 are provided as a Source Data file, available as a Supplementary Information file. A reporting summary for this Article is available as a Supplementary Information file. All unique materials are available on reasonable request from the corresponding authors.
