# Supplementary material for: A five-residue motif for the design of domain swapping in proteins
Source: Nat Commun. 2019 Jan 28;10:452. doi: 10.1038/s41467-019-08295-x (PMC6349918; doi:10.1038/s41467-019-08295-x)
Supplement: Supplementary file 1 — Supplementary Information [file 41467_2019_8295_MOESM1_ESM.pdf]

Supplementary Information for

A five-residue motif for the design of domain swapping in proteins

Neha Nandwani, Parag Surana, Hitendra Negi, Nahren M. Mascarenhas, Jayant B. Udgaonkar*, Ranabir Das* and Shachi Gosavi*

This file includes:

Supplementary Figures 1-22

Supplementary Tables 1-8

Supplementary Methods

Supplementary References

*Correspondence to: S.G. (shachi@ncbs.res.in) or R.D. (rana@ncbs.res.in) or J.B.U. (jayant@ncbs.res.in or jayant@iiserpune.ac.in)

<u>**Supplementary Figures**</u>

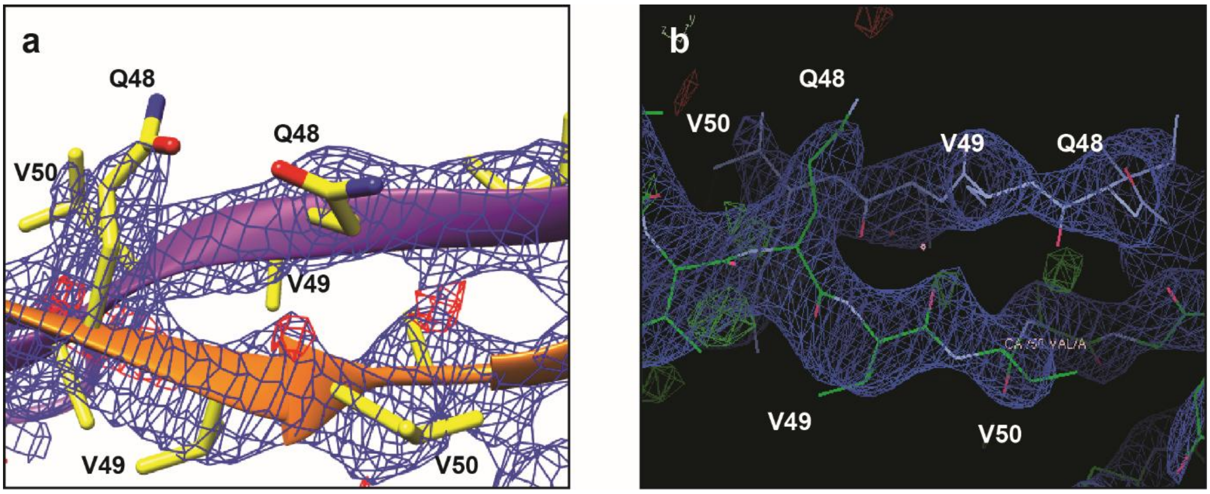

Supplementary Figure 1. 2Fo-Fc and Fo-Fc maps of the QVVAG motif in L1MN (a) The 2Fo-Fc simulated anneal electron density omit map is contoured at 1.6σ (blue) and the Fo-Fc electron density map (red) contoured at 2.8σ. The figure was made in UCSF Chimera. **(b)** A snapshot from the same figure in Coot is shown, wherein the Fo-Fc electron density is shown as a difference map (positive: Green and negative: Red).

a

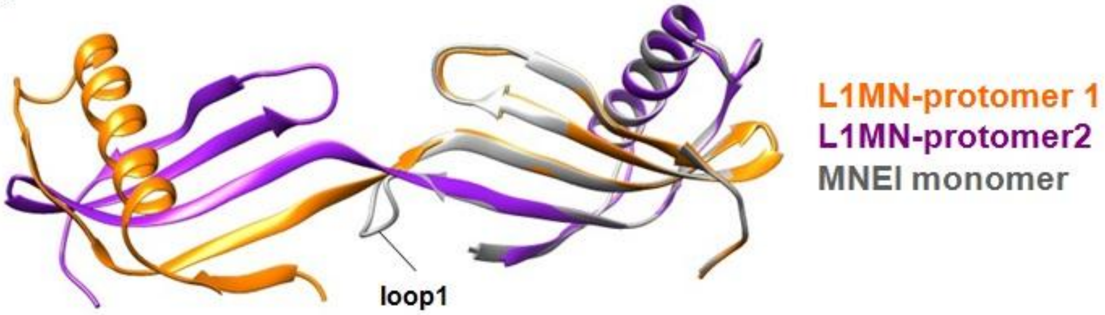

b

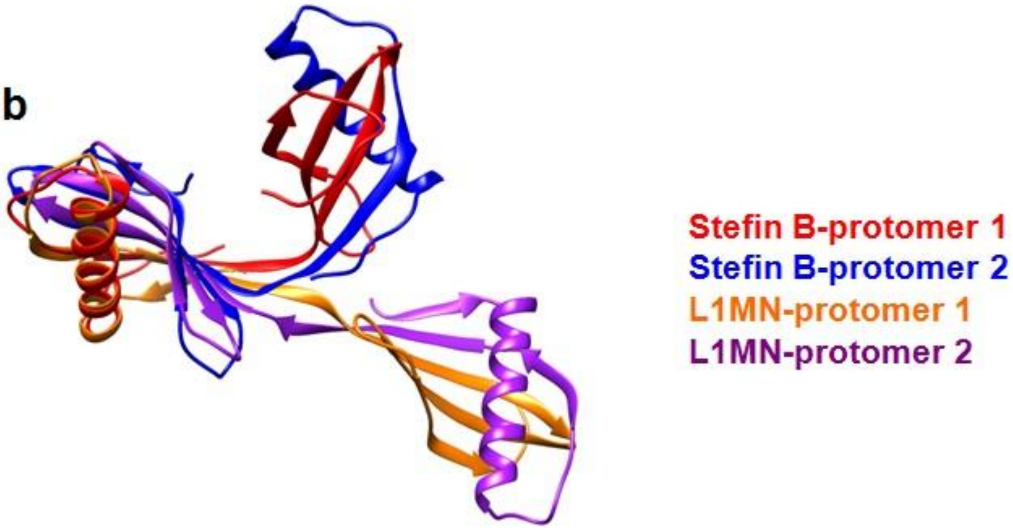

Supplementary Figure 2. Structural overlap of the L1MN domain-swapped dimer with wt MNEI and the Stefin B domain-swapped dimer (a) Superposition of one of the subunits of the L1MN swapped dimer (two protomers shown in orange and purple) with wt MNEI monomer (gray) is shown. The structures superpose with an rmsd of 1.2 Å. **(b)** Superposition of L1MN (two protomers in orange and purple) with Stefin B (two protomers shown in red and blue, PDB ID 2OCT) domain-swapped dimer shows that the relative orientation between the functional units in the two proteins is different.

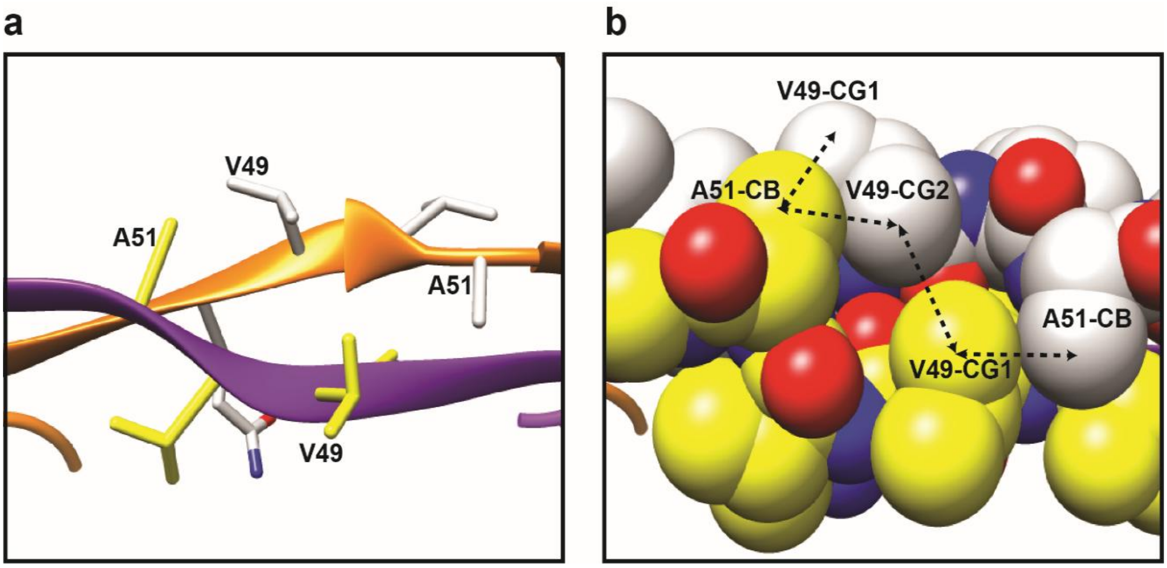

Supplementary Figure 3. Hydrophobic interactions between the QVVAG motifs in L1MN (a) The orientation of the hydrophobic residues V49 and A51 at loop1 in L1MN is shown. The ribbon colors are orange and purple for protomer 1 and 2, respectively. The side chain carbon atoms are colored in white and yellow for protomer 1 and 2, respectively. Nitrogen atoms are colored blue and oxygen atoms are colored red. **(b)** Same as (a) except that ribbons are absent and atoms are shown as spheres. The color code is same as (a). The van der Waals contacts are shown as black dotted lines with arrows, and the atoms forming these contacts are labeled in black. See supplementary Table 3 for a complete list of contacts.

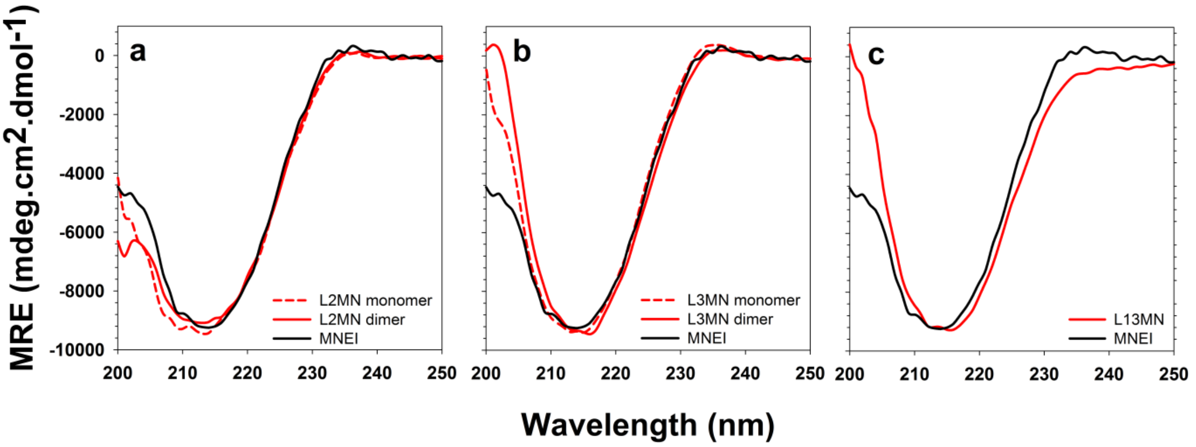

Supplementary Figure 4. Secondary structure is conserved in different MNEI loop mutants Far-UV CD spectra monitored at pH 7 for **(a)** L2MN monomer and dimer, **(b)** L3MN monomer and dimer, and **(c)** L13MN are shown. For the L2MN and L3MN variants, the monomer and dimer were separated by SEC. The CD spectrum for wt MNEI is shown for comparison in each panel. Source data are provided as a Source Data file.

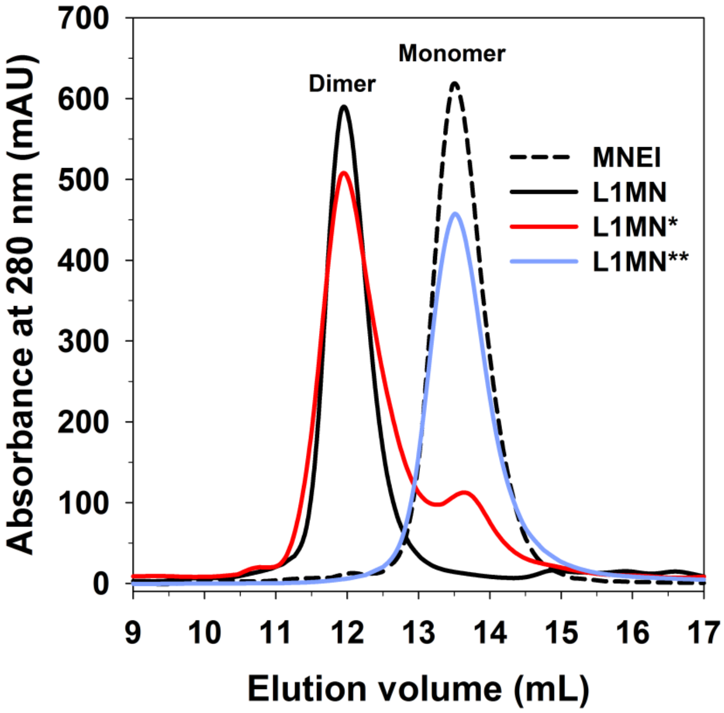

Supplementary Figure 5. Stabilization of the monomeric conformation of L1MN A comparison of the SEC profiles of L1MN (QVVAG), L1MN* (QVNAG) and L1MN** (QNNAG) is shown. The SEC profile of wt MNEI is shown for comparison. Source data are provided as a Source Data file.

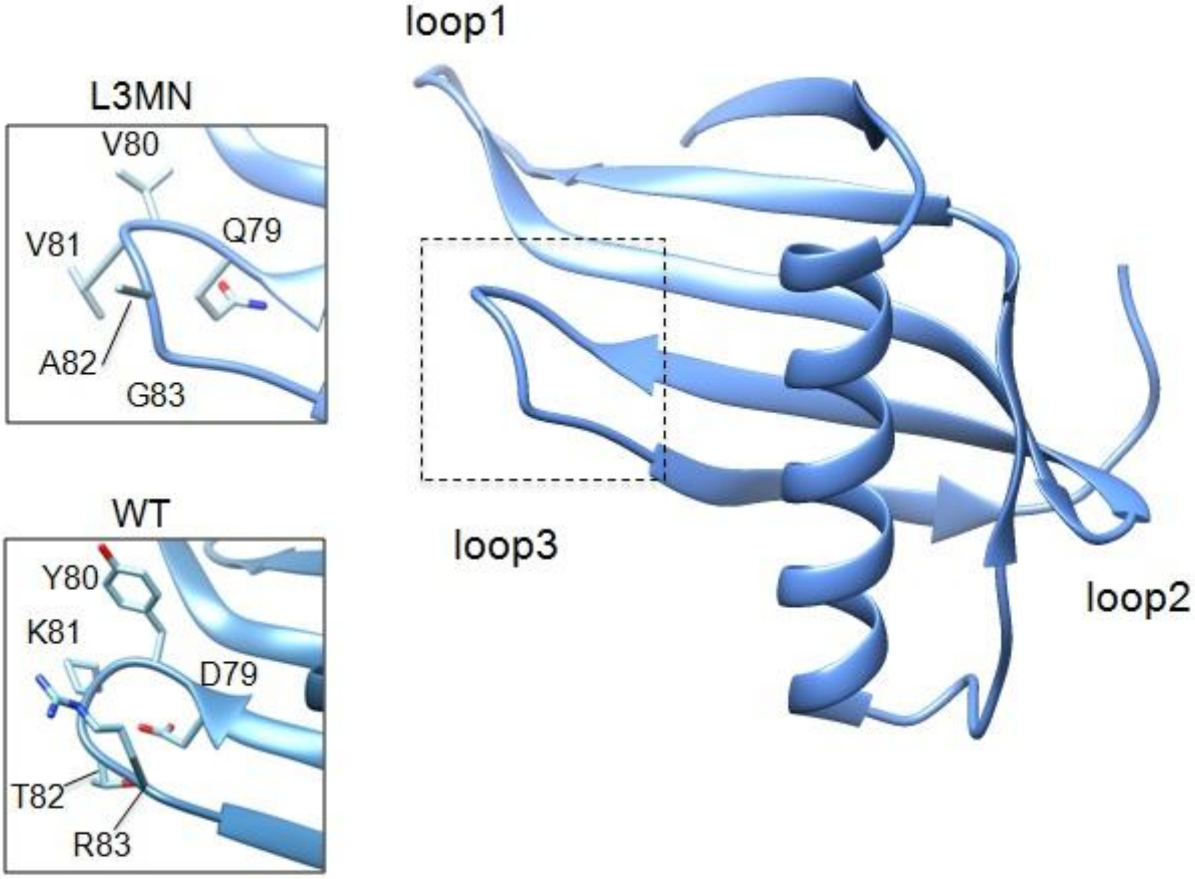

Supplementary Figure 6. Crystal structure of the L3MN monomer The structure of L3MN monomer solved by X-ray crystallography is shown. The loop3 residues are expanded and shown for L3MN and the wt MNEI.

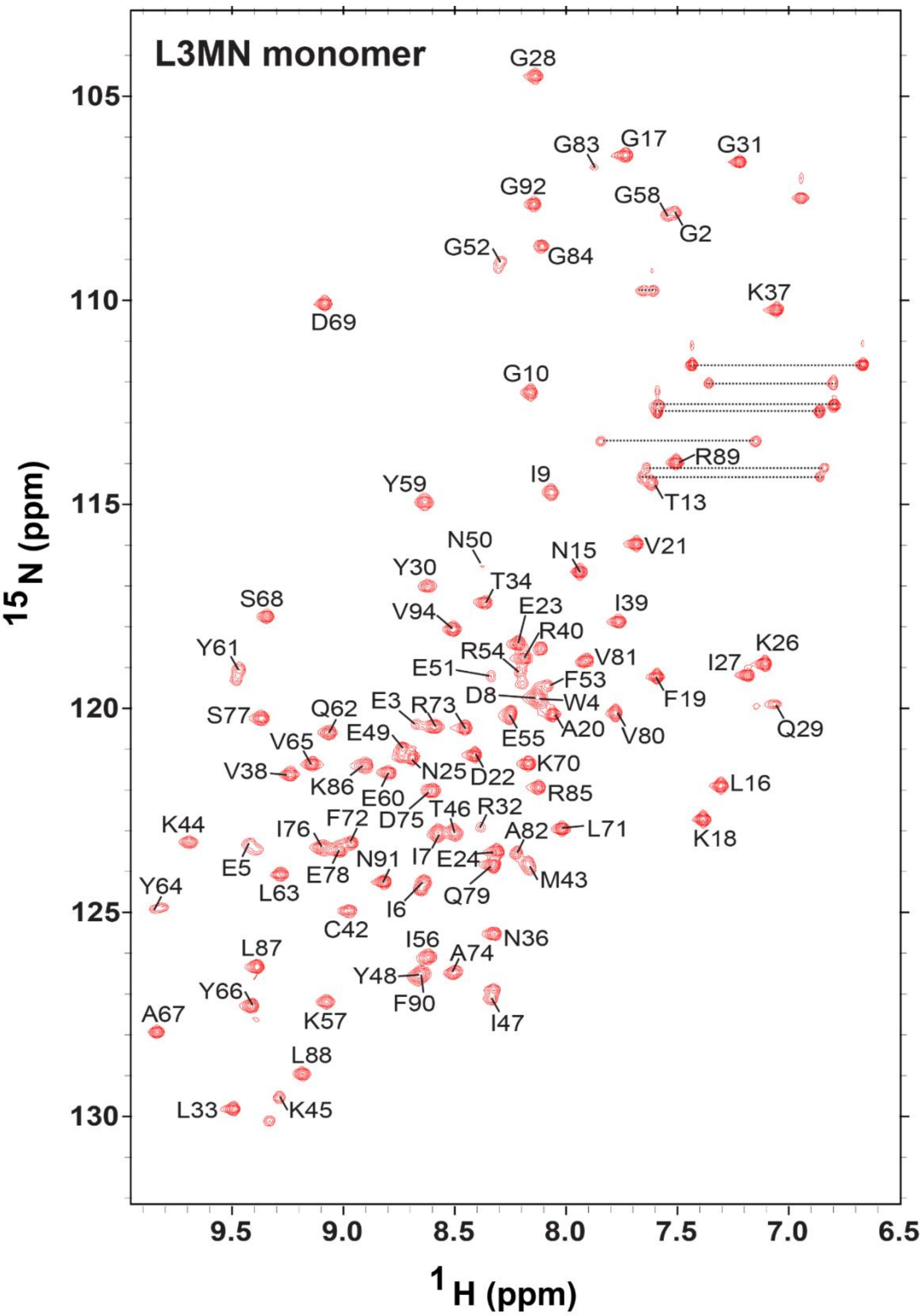

Supplementary Figure 7. Assignment of backbone chemical shifts in the L3MN monomer ^{15}N-HSQC spectrum of the L3MN monomer is shown. The assigned backbone amide resonances are labeled.

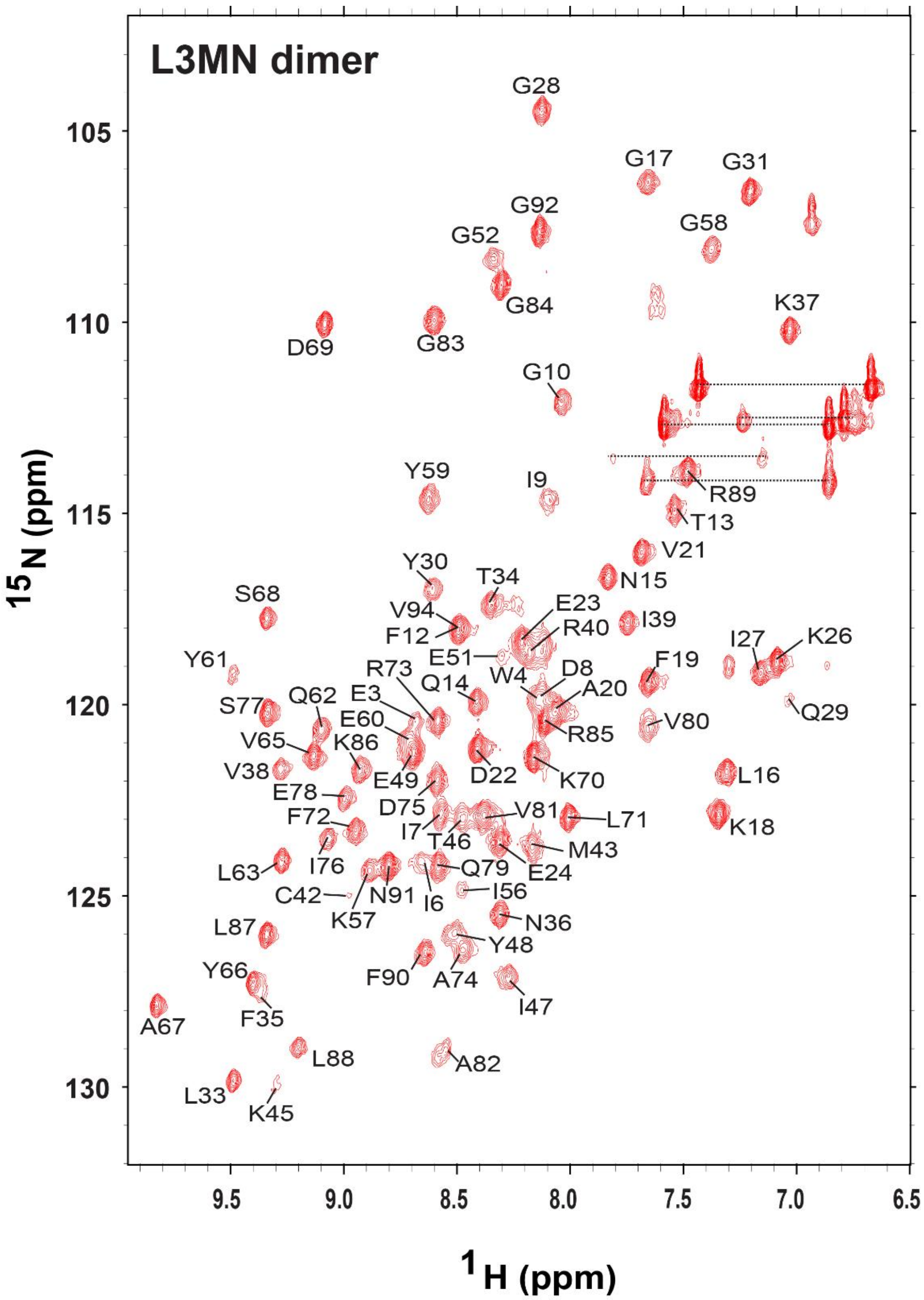

Supplementary Figure 8. Assignment of backbone chemical shifts in the L3MN dimer ^{15}N-HSQC spectrum of the L3MN dimer is shown. The assigned backbone amide resonances are labeled.

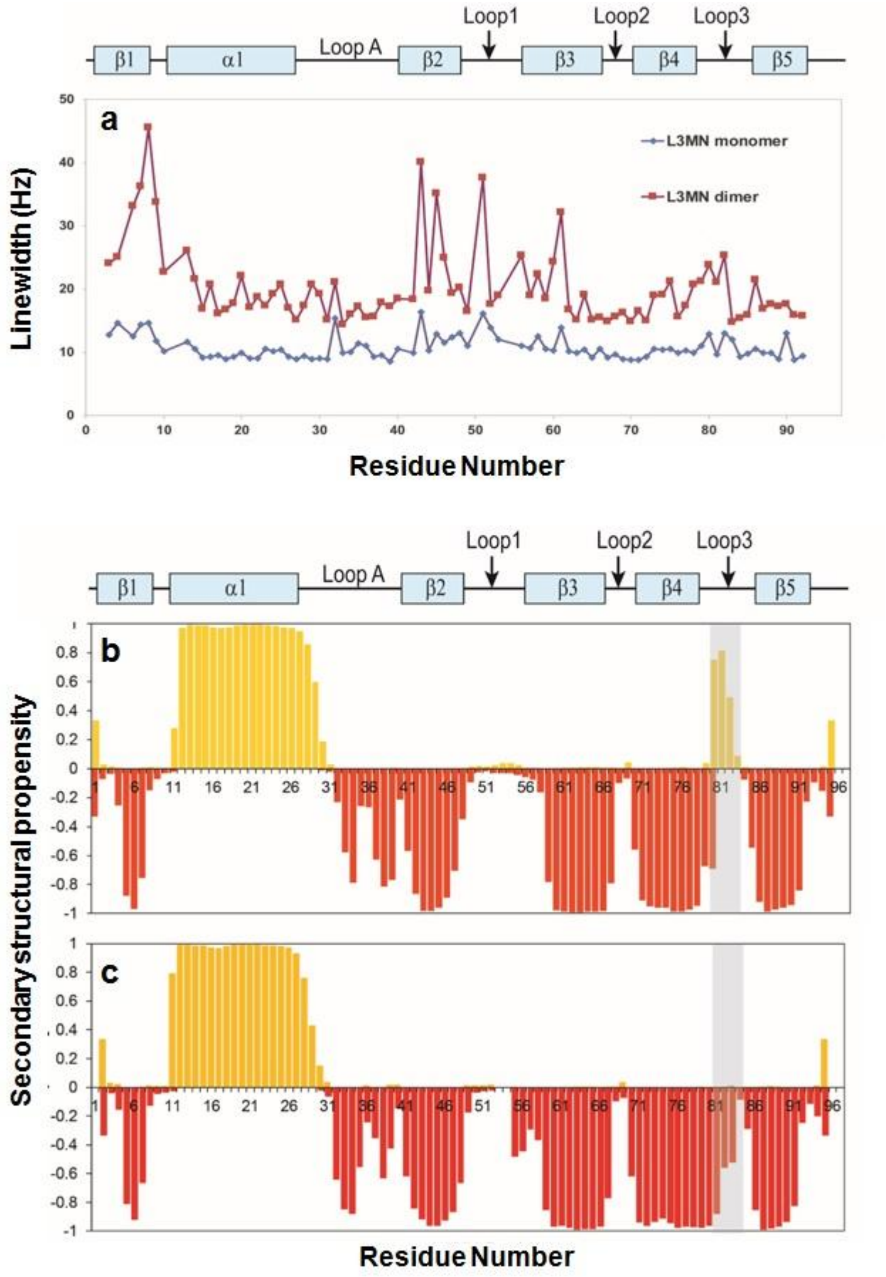

Supplementary Figure 9. Characterization of the L3MN monomer and dimer by NMR **(a)** Line-widths of the amide resonance peaks in the ^{15}N-HSQC spectra of the L3MN monomer and dimer are shown. **(b)** Secondary structure prediction of the L3MN monomer obtained by analyzing the backbone and $^{13}C_{\beta}$ chemical shifts by TALOS+[1]. Positive values indicate α-helices and negative values indicate β-strands. The gray box highlights the residues 80-83. **(c)** Similar analysis for the L3MN dimer as in (b). The linear arrangement of the secondary structural elements of MNEI is indicated on the top for reference in each panel. Source data are provided as a Source Data file.

a

Expected Φ/ψ angles				
	Φ(81)	Ψ(81)	Φ(82)	Ψ(82)
β-hairpin turn I	-60	-30	-90	0
β-strand	-120	+120	-120	+120
**NMR derived Φ/ψ angles**				
	Φ(81)	Ψ(81)	Φ(82)	Ψ(82)
L3MN monomer	-67	-28	-91	-5
L3MN dimer	-116	+136	-106	+135

b

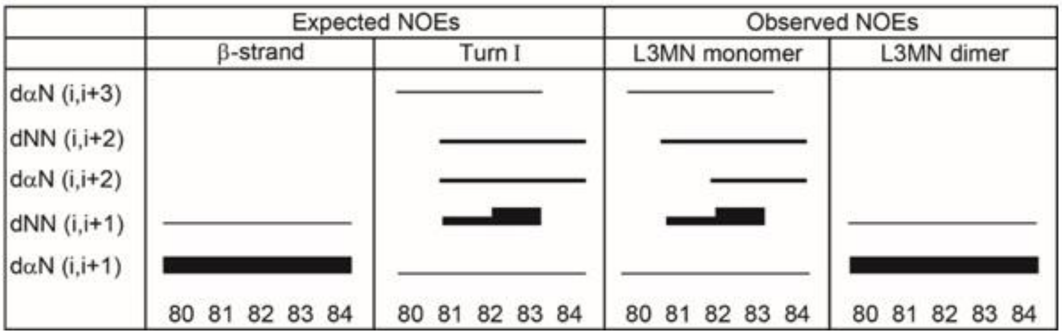

Supplementary Figure 10. Differential secondary structure of loop3 in the L3MN monomer and dimer (a) The ϕ, ψ angles obtained from the NMR data for residues 81 and 82 in loop3 of L3MN monomer and dimer are tabulated. For comparison, the expected ϕ, ψ angles of the 81-82 region, when present in a β-hairpin or a β-strand conformation, are provided. **(b)** The typical NOE pattern for a β-hairpin and a β-strand are given in the left half of the table. The observed NOEs in the L3MN monomer and dimer in the region 80-84 are given on the right. The thickness of the line is proportional to the intensity of the observed NOE peak.

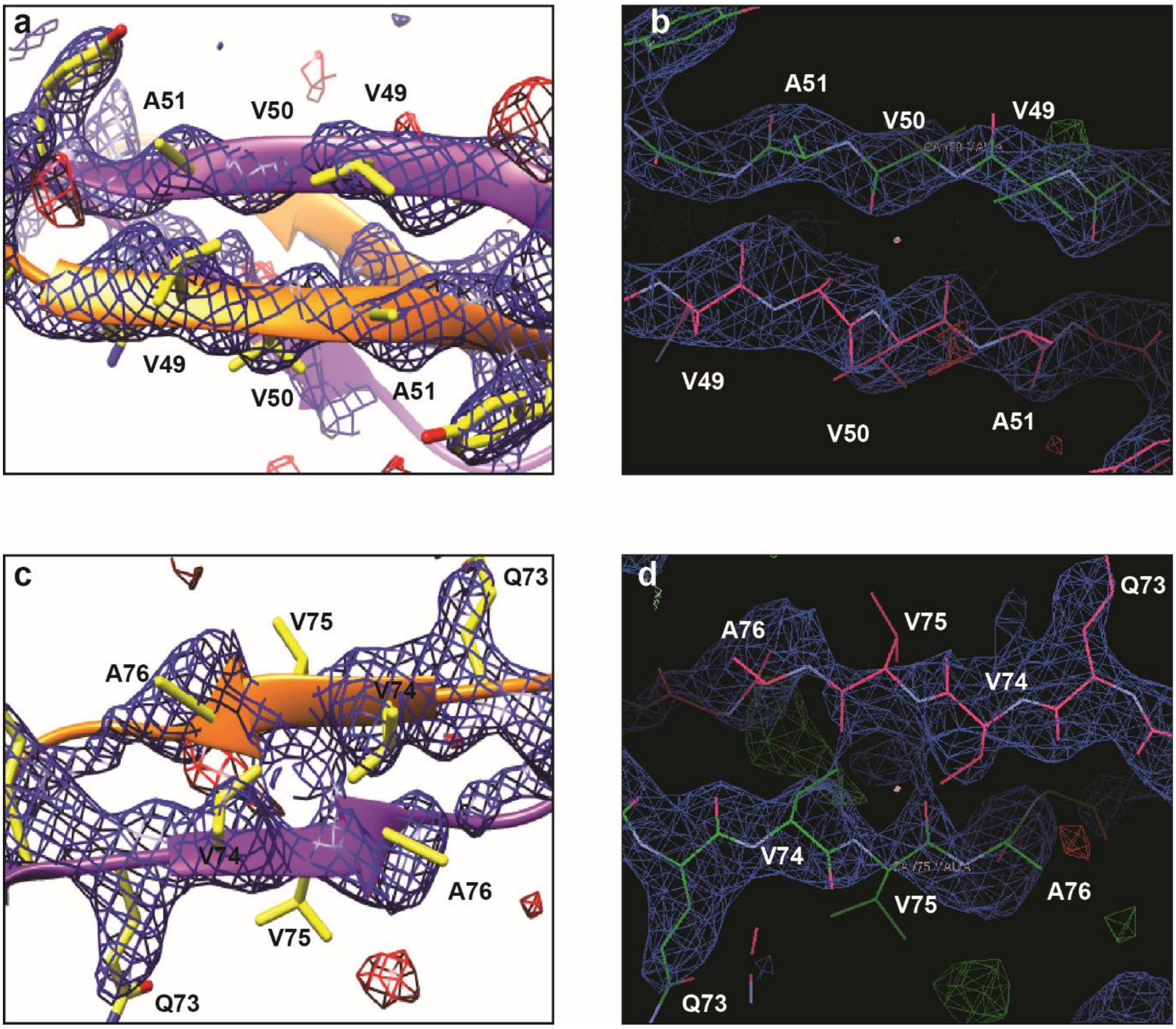

Supplementary Figure 11. 2Fo-Fc and Fo-Fc maps of the QVVAG motif in L13MN (a) The 2Fo-Fc simulated anneal electron density omit map contoured at 1.6σ (blue), and the Fo-Fc electron density map (red) contoured at 2.8σ for loop1 is shown. The figure was made in UCSF Chimera with secondary structure shown with ribbons. **(b)** A snapshot of the same figure in Coot, where the Fo-Fc electron density is shown as a difference map (positive: Green and negative: Red). **(c)** and **(d)** are same as (a) and (b), respectively, but for the loop3 QVVAG motif.

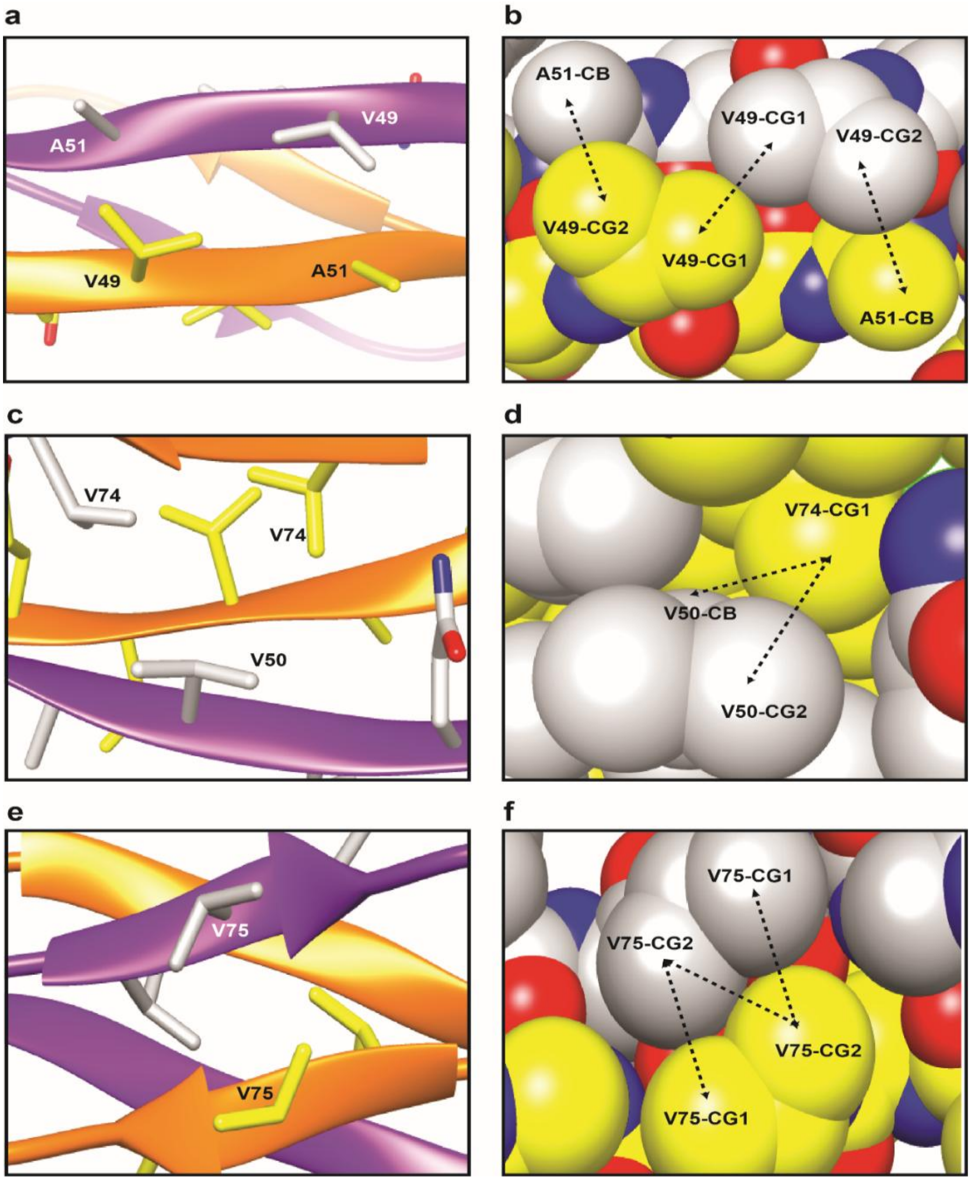

Supplementary Figure 12. Hydrophobic interactions between the QVVAG motifs at loop1 and loop3 in L13MN (a) The orientation of the hydrophobic residues V49 and A51 at the loop1 secondary interface is shown. The side chain carbon atoms are colored in white and yellow for protomer 1 and 2, respectively. Nitrogen atoms are colored blue and oxygen atoms are colored red. **(b)** is same as (a) except that ribbons are absent and atoms are shown as spheres. **(c)** and **(d)** show the side chains in stick and sphere representation, respectively, in the region between loop1 and loop3. **(e)** and **(f)** show the side chains in stick and sphere representation, respectively, at the loop3 interface. In (b), (d) and (f), the van der Waals contacts are shown as black dotted lines with arrows, and the atoms forming these contacts are labeled in black. See Supplementary Table 4 for a complete list of contacts.

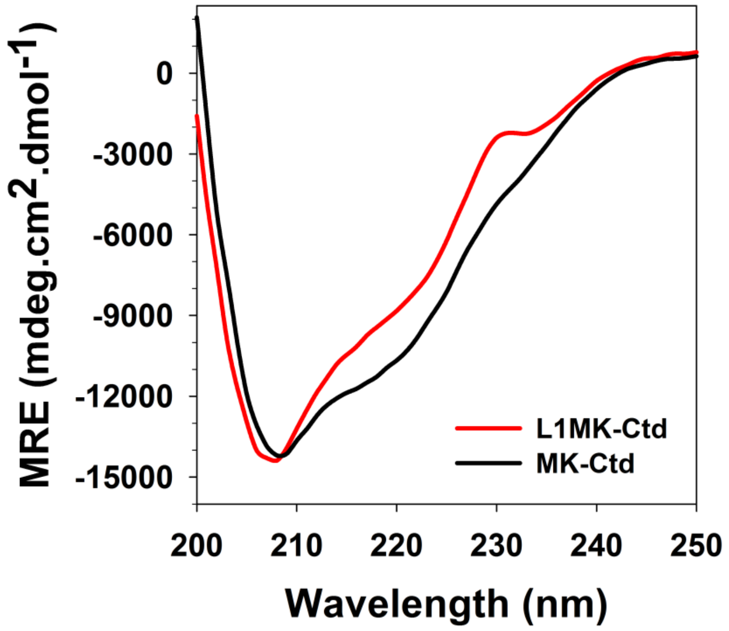

Supplementary Figure 13. Secondary structure is conserved in L1MK-Ctd Far-UV CD spectra monitored at pH 8 for the dimeric L1MK-Ctd is shown. The CD spectrum of the monomeric wt MK-Ctd is shown for comparison. Source data are provided as a Source Data file.

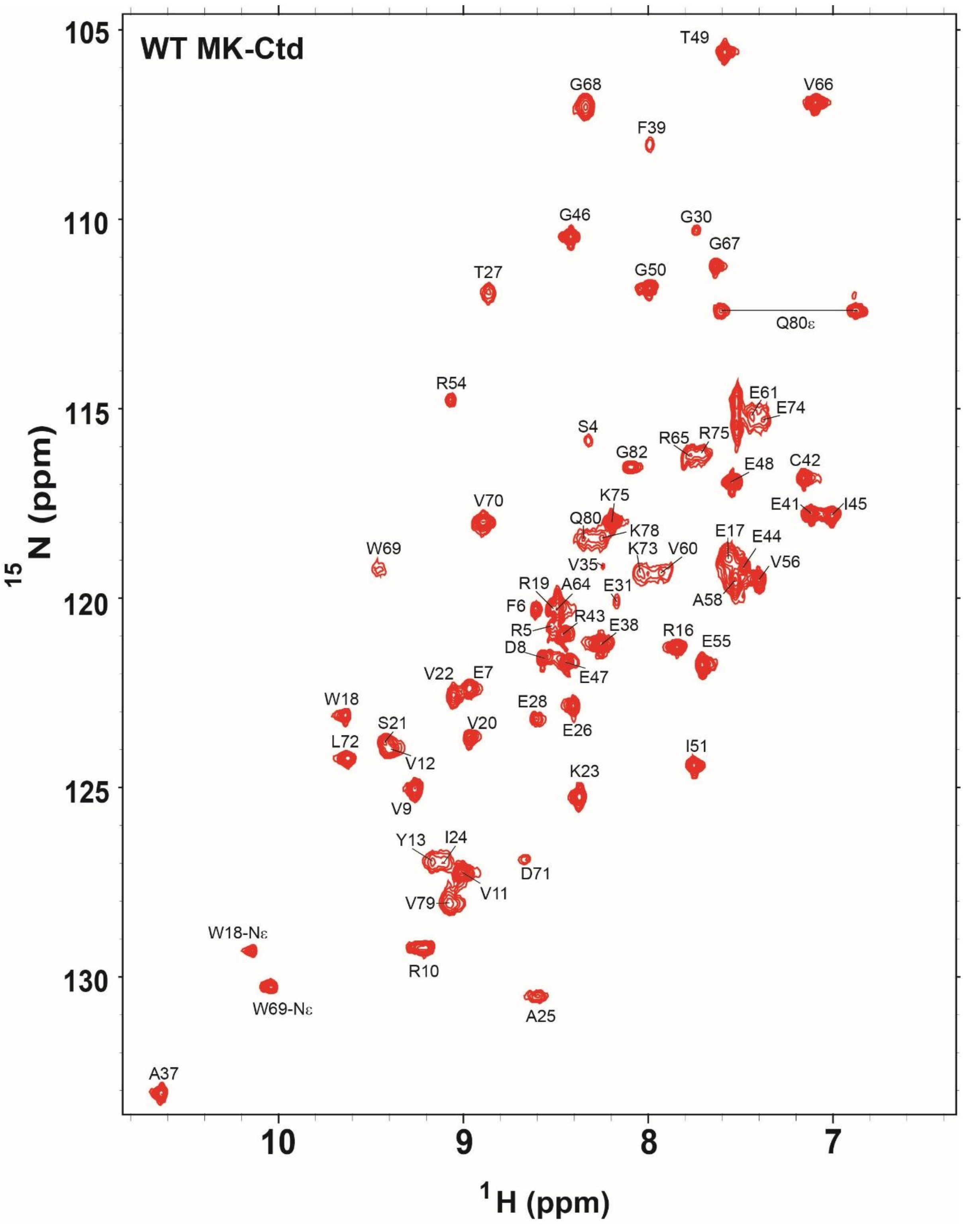

Supplementary Figure 14. Assignment of backbone chemical shifts in the monomeric wt MK-Ctd ^{15}N-HSQC spectrum of the monomeric wt MK-Ctd is shown. The assigned backbone amide resonances are labeled.

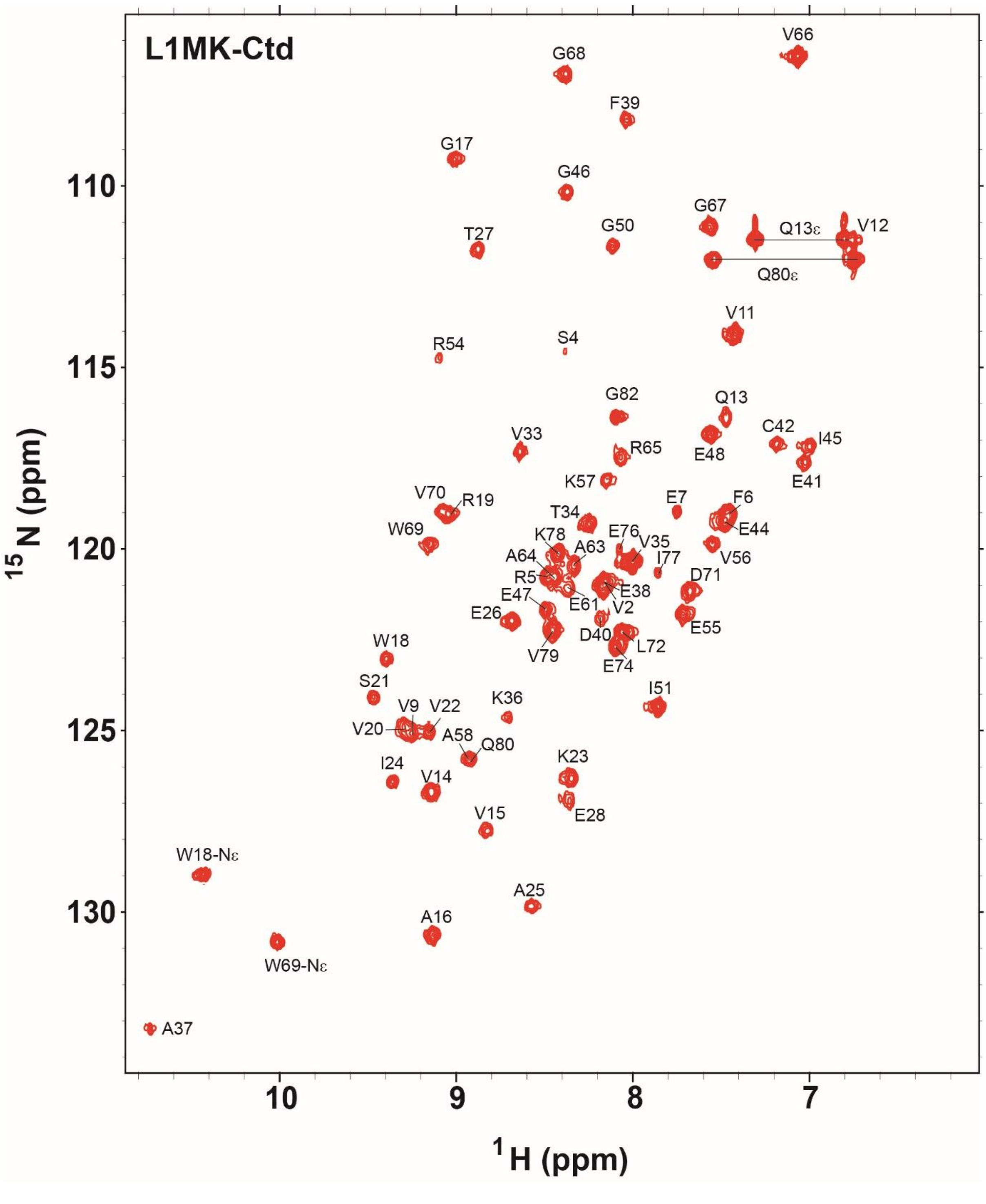

Supplementary Figure 15. Assignment of backbone chemical shifts in the dimeric L1MK-Ctd ^{15}N-HSQC spectrum of the L1MK-Ctd dimer is shown. The assigned backbone amide resonances are labeled.

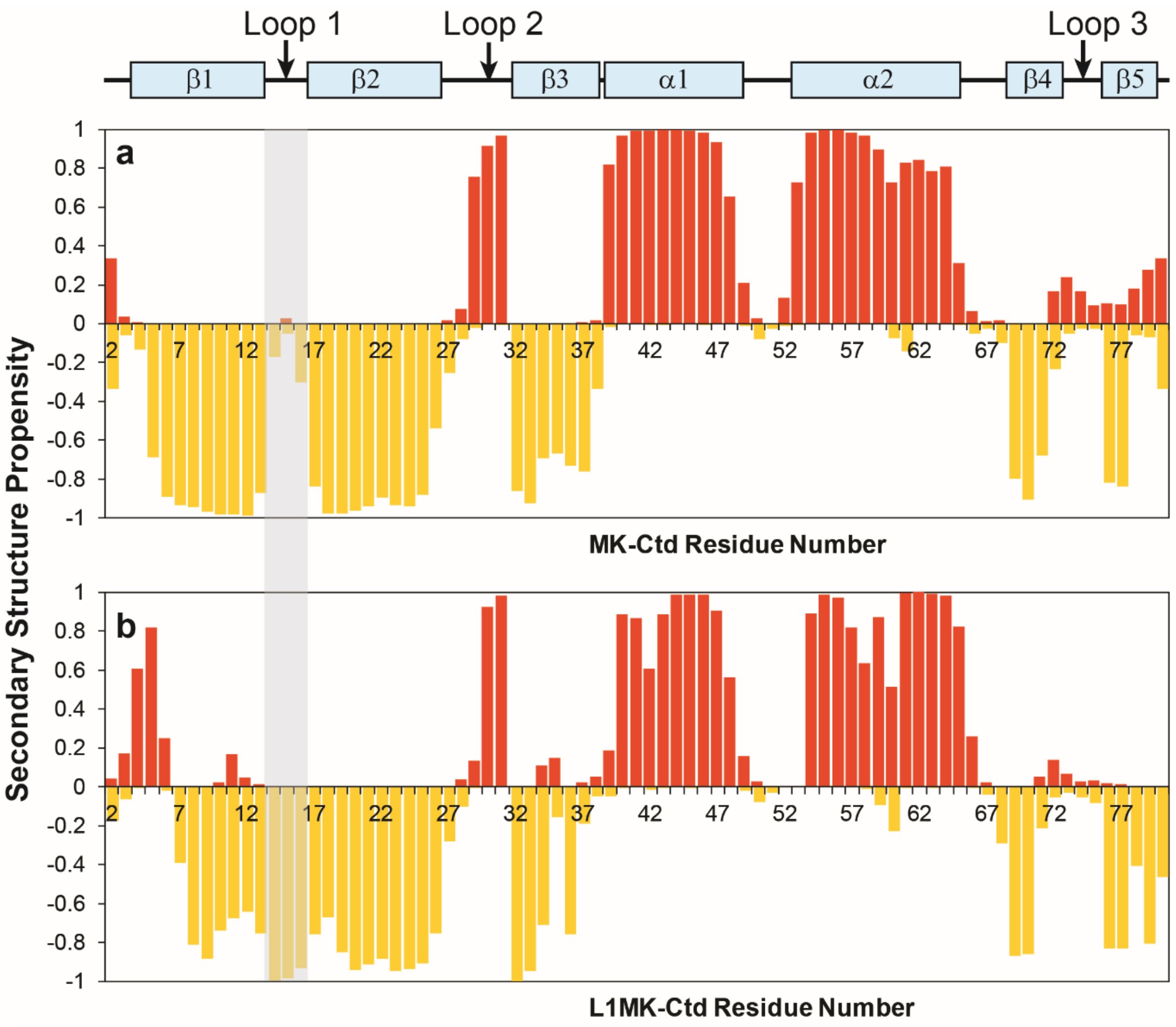

Supplementary Figure 16. Secondary structure analysis of MK-Ctd and L1MK-Ctd by NMR. (a) Secondary structure prediction of the monomeric wt MK-Ctd, obtained by analyzing the backbone and $^{13}C_{\beta}$ chemical shifts by TALOS+[1], is shown. Positive values indicate α-helices and negative values indicate β-strands. The gray box highlights the residues 14-16. **(b)** Similar analysis for the L1MK-Ctd dimer as in (a). The linear arrangement of the secondary structural elements of MK-Ctd is indicated on the top for reference. Source data are provided as a Source Data file.

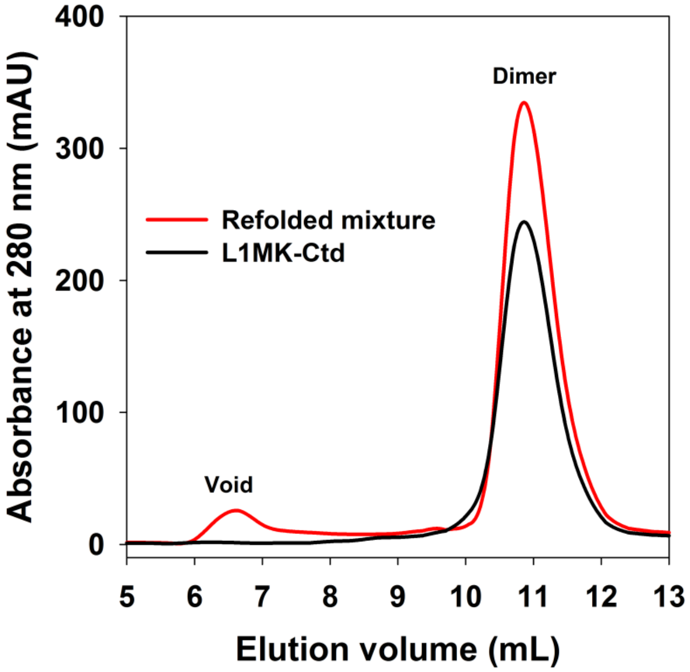

Supplementary Figure 17. SEC profile of refolded L1MK-Ctd The SEC profile of the refolded mixture prepared for inter-chain NOE experiments is shown. More than 90% of the total protein in the refolded mixture was found to be dimeric. The SEC profile of L1MK-Ctd dimer is shown for comparison. L1MK-Ctd dimer was similarly incubated overnight in 0.5 M GdnHCl containing buffer. Source data are provided as a Source Data file.

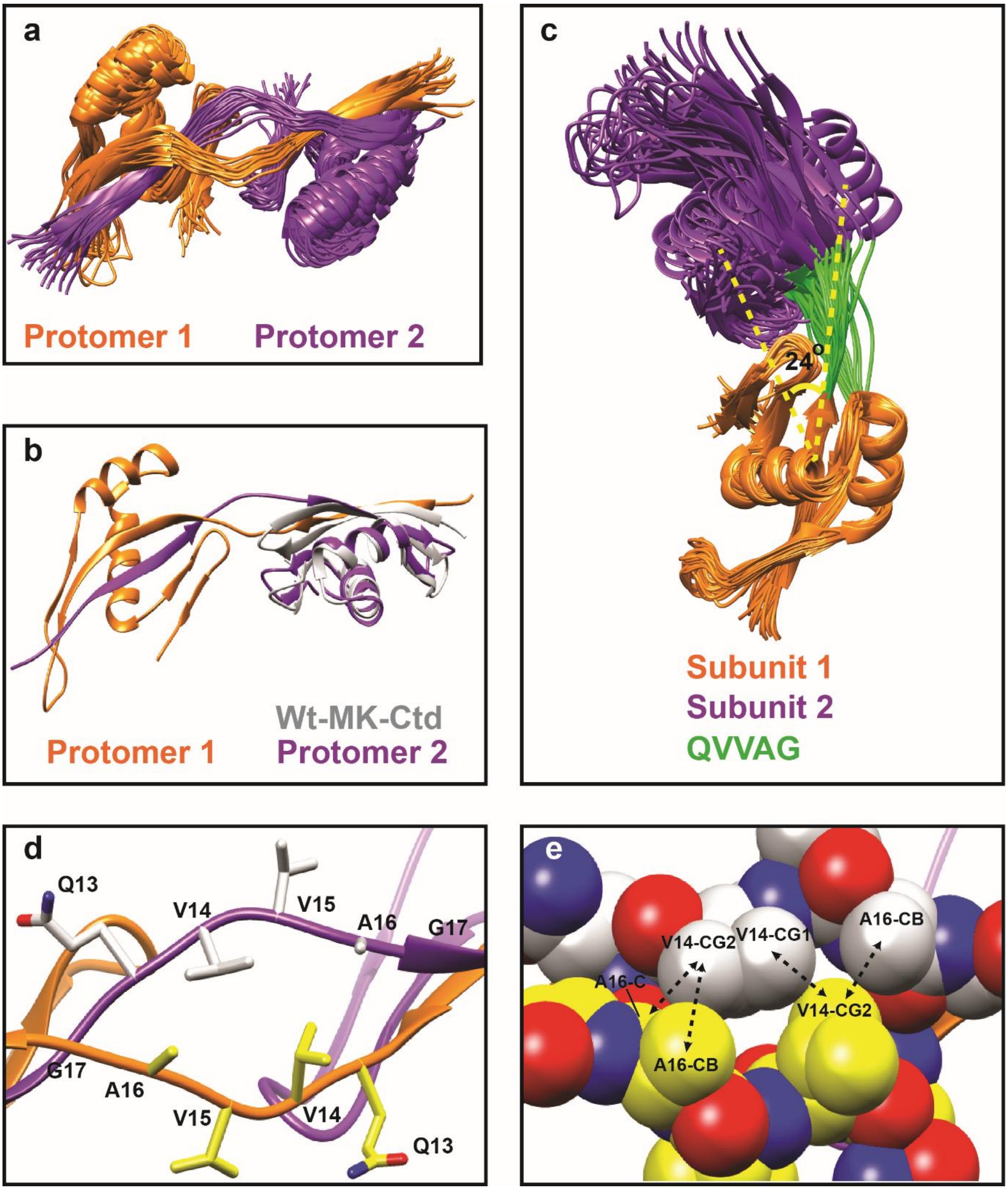

Supplementary Figure 18. NMR structure of the L1MK-Ctd dimer and the hydrophobic interactions at the secondary interface (a) The twenty lowest energy NMR structures of L1MK-Ctd dimer are shown. The two protomers are colored in orange and purple. **(b)** A comparison of the structures of the monomeric wt MK-Ctd and dimeric L1MK-Ctd is shown, where the wt MK-Ctd structure (PDB ID: 3C19, residues 98-178) is superimposed with one of the subunits of the L1MK-Ctd dimer. **(c)** An alignment of the lowest energy structures using residues of subunit 1, which includes the N-terminal β1 strand

from protomer 2 and the rest (β2-β3-α1-α2-β4-β5) from protomer 1, except for the hinge region. Here the subunit 1 is colored orange, subunit 2 is colored purple, and the hinge region is colored green. The range of conformations was measured by calculating the angle between the center of the mass within each subunit, which is shown here. **(d)** The orientation of the QVVAG motif residues in loop1 of L1MK-Ctd are shown. The ribbons colors are orange and purple for protomer 1 and 2, respectively. The side chain carbon atoms are colored in white and yellow for protomer 1 and 2, respectively. Nitrogen atoms are colored blue and oxygen atoms are colored red. **(e)** Same as (d) except that ribbons are absent and atoms are shown as spheres. The color code is same as in (d). The van der Waals contacts are shown as black dotted lines with arrows, and the atoms forming these contacts are labeled in black. See Supplementary Table 7 for a complete list of contacts.

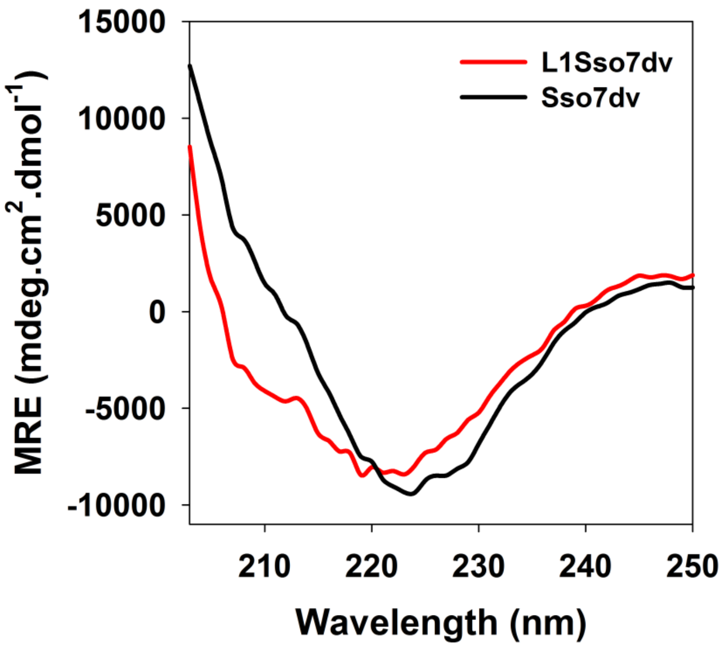

Supplementary Figure 19. Secondary structure of L1Sso7dv Far-UV CD spectra monitored at pH 7.5 for L1Sso7dv is shown. The CD spectrum for the monomeric Sso7dv is shown for comparison. Source data are provided as a Source Data file.

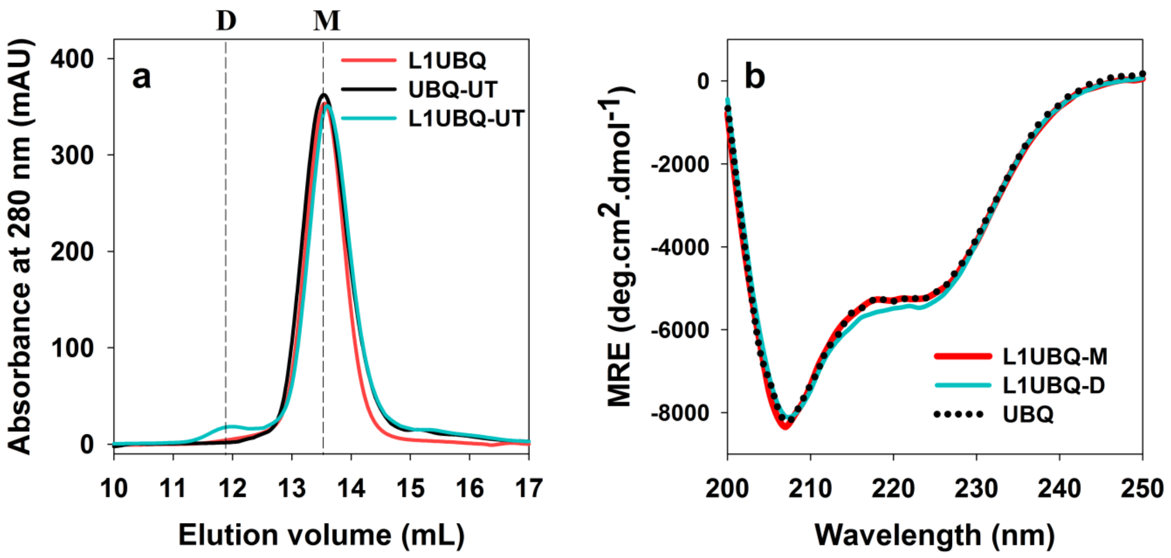

Supplementary Figure 20. Engineered domain swapping in L1UBQ (a) Size exclusion profile of the loop1 variant of UBQ, before (L1UBQ) and after urea treatment (L1UBQ-UT), at pH 8.3 is shown. The SEC profile of the urea-treated wt UBQ (UBQ-UT) is shown for comparison. The elution volumes of the monomer (M) and dimer (D) are indicated. **(b)** Far-UV CD spectra monitored at pH 8.3 for the monomeric (L1UBQ-M) and dimeric (L1UBQ-D) fractions of urea-treated L1UBQ are shown. The spectra were acquired immediately after the monomeric and dimeric fractions of L1UBQ were separated by SEC. The CD spectrum for wt UBQ is shown for comparison. Source data are provided as a Source Data file.

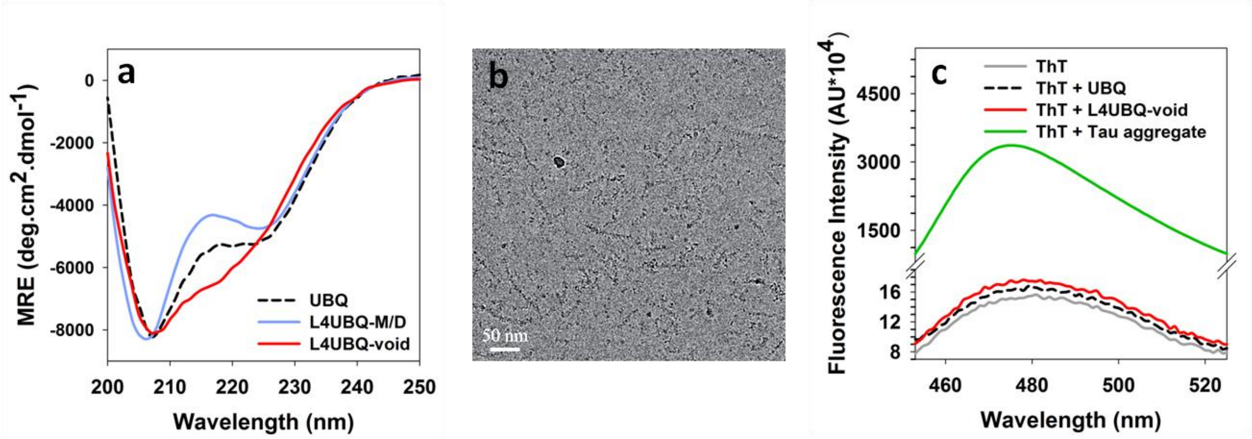

Supplementary Figure 21. Engineered domain swapping in L4UBQ (a) Far-UV CD spectra monitored at pH 8.3 for the monomer-dimer mixture (L4UBQ-M/D; interconversion between the monomer and dimer after separation by SEC was found to be fast) and void fraction (L4UBQ-void) of purified L4UBQ are shown. The various oligomeric fractions of L4UBQ were separated by SEC immediately before acquisition of the spectra. The CD spectrum for wt UBQ is shown for comparison. The observed deviation in the CD spectrum of L4UBQ-void from the wt spectrum at around 216 nm can be attributed to possible structural rearrangements that may have occurred upon multimerization, including the possibility of the conversion of the long loop between β4-β5 (Figure 6c) to a β-strand like conformation in the oligomer. A similar change in the CD spectrum has been observed earlier for the domain-swapped trimer of the loop-insertion mutant of chymotrypsin inhibitor 2[2]. **(b)** A micrograph of the void fraction of L4UBQ on ice showing elongated fibril-like structures is shown. These structures are variable in length, some of which tend to curve. The length of the fibrils varies between 36-85 nm (scale bar: 50 nm). The big dark objects are contaminants from ice. **(c)** The fluorescence emission spectrum of thioflavin T (ThT), when mixed with either wt UBQ (ThT + wt UBQ), or the oligomer eluting in the void fraction for L4UBQ (ThT + L4UBQ-void) collected from the SEC profile showed in (b) are shown. As a positive control, the fluorescence emission spectrum of ThT, when mixed with the β-rich amyloid aggregate of Tau K18[3] is shown. Binding to β-rich oligomers resulted in ~200-fold increase in the fluorescence of ThT. The fluorescence emission spectrum of ThT alone is shown for comparison. Source data are provided as a Source Data file.

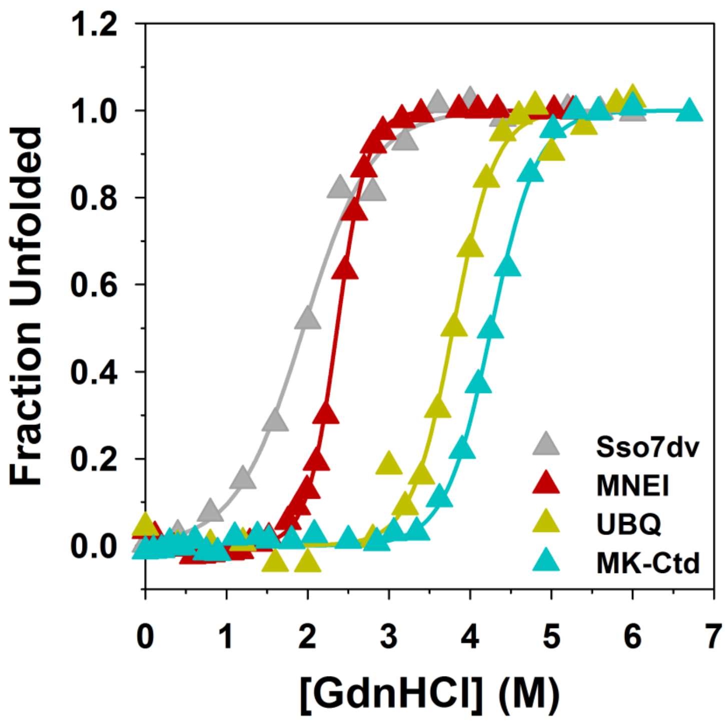

Supplementary Figure 22. Stability of the target proteins The equilibrium unfolding transitions of wt Sso7dv, wt MNEI, wt UBQ and wt MK-Ctd at pH 7, 25°C as monitored by far-UV CD at 222 nm are shown. Fit to a two-state model[4] yielded values for ΔG_u of 2.8 ± 0.5 kcal mol^{-1} for Sso7dv, 6.9 ± 0.2 kcal mol^{-1} for MNEI, 7.8 ± 0.3 kcal mol^{-1} for UBQ, and 8.9 ± 0.2 kcal mol^{-1} for MK-Ctd. The results are presented as mean ± s.d.; the error reported here is the fitting error. The experiment was done once, the values reported here are in agreement with the previously reported values for these proteins[5–8]. The data for MK-Ctd is reproduced from the Ph. D. thesis of Hitesh Rafalia titled "*To study the mechanistic details of folding/unfolding reaction of A98-178 domain of MK0293*" submitted to Manipal Academy of Higher Education, Madhavnagar, Manipal-576104, India. Source data are provided as a Source Data file.

Supplementary Tables

Supplementary Table 1. Apparent molar masses of different loop variants estimated from multi-angle light scattering experiments

Loop variant	Molecular weight (kDa)
**monomeric BSA (control)**	66.5 ± 4.6
**wt MNEI**	13.2 ± 1.5
**L1MN**	23.2 ± 2.4
**L2MN dimer**	26.5 ± 2.5
**L3MN dimer**	24.4 ± 2.1
**L13MN**	23.2 ± 1.8
**wt MK-Ctd**	10.8 ± 1.1
**L1MK-Ctd**	22.5 ± 2.3
**wt UBQ**	8.6 ± 0.9
**L4UBQ dimer**	16.3 ± 2.1
**L4UBQ void**	353.4 ± 5.5

The results are presented as mean ± s.d.; s.d. was calculated by the software Astra from at least 10 different measurements of the same sample.

Supplementary Table 2. X-ray crystallographic data collection and refinement statistics

	L1MN	L13MN	L3MN-monomer
**Data Collection**			
Wavelength (Å)	0.98	0.97	1.54
Resolution range (Å)	43.1 - 2.3 (2.4 - 2.3) [a]	41.7 - 2.3 (2.4 - 2.3)	24.0 - 1.8 (1.9 - 1.8)
Space group	C 1 2 1	P 31 2 1	P 1 21 1
Unit cell dimensions:			
a, b, c (Å)<br>α, β, γ	108.6, 86.3, 86.8,<br>90°, 127.5°, 90°	48.1, 48.1, 103.5,<br>90°, 90°, 120°	35.6, 48.1, 62.7<br>90°, 103.7^o, 90°
Total reflections	86249 (6559)	38571 (3926)	83697 (4811)
Unique reflections	26814 (2522)	6521 (642)	17514 (1552)
Multiplicity	3.2 (2.6)	5.9 (6.1)	4.8 (3.1)
Completeness (%)	97.2 (91.5)	97.3 (100.00)	98.8 (89.0)
Mean I/sigma(I)	15.0 (0.9)	22.8 (2.5)	24.0 (3.1)
Wilson B-factor ($Å^2$)	77.6	71.5	32.3
Reflections used for R-free	1367	646	885
**Refinement**			
R_{work}	0.2 (0.4)	0.2 (0.4)	0.2 (0.3)
R_{free}	0.3 (0.4)	0.3 (0.4)	0.2 (0.3)
**No. of atoms**	3694	724	1677
Protein	3645	720	1523
ligands	0	0	0
water	49	4	154
**No. of Protein residues**	449	90	187
**R.m.s deviations**			
bond length (Å)	0.007	0.006	0.010
angles (°)	1.3	1.0	1.2
**Ramachandran Plot and MolProbity**[9]			
% favoured	93.0	93.0	95.0
% allowed	7.0	7.0	4.5
% outliers	0	0	0.5
Clashscore	5.5	1.4	4.3
**Average B-factor ($Å^2$)**			
Model (all atoms)	98.7	81.5	41.8
Protein	98.9	81.6	41.6

Ligands	0	0	0
Solvent	88.3	74.1	43.8
**PDB ID**	5YCU	5YCW	5YCT

[a]Statistics for the highest-resolution shell are shown in parentheses.

Supplementary Table 3. van der Waals contacts and hydrogen bonds observed between the two protomers in L1MN at the QVVAG motif.

**van der Waals Contacts at loop 1**						
Atom in Protomer 1			Atom in Protomer 2			
Residue	Residue No.	Atom	Residue	Residue No.	Atom	Distance (Å)
GLN	48	OE1	GLN	48	OE1	2.8
VAL	49	CG1	ALA	51	CB	3.4
VAL	49	O	GLN	48	CA	3.0
VAL	49	CG2	ALA	51	CB	3.6
ALA	51	CB	VAL	49	CG1	3.7
VAL	49	O	GLN	48	CB	3.3
ALA	51	CB	VAL	49	CB	4.0
VAL	49	O	VAL	49	O	3.1
VAL	49	O	GLN	48	C	3.4
VAL	49	CG2	VAL	49	CG1	4.1
VAL	49	CB	ALA	51	CB	4.1
**Hydrogen Bonds at loop 1**						
Atom in Protomer 1			Atom in Protomer 2			
Residue	Residue No.	Atom	Residue	Residue No.	Atom	Distance (Å)
VAL	49	N	VAL	49	O	2.9
VAL	49	O	VAL	49	N	2.8
ALA	51	N	ILE	47	O	2.6
ILE	47	O	ALA	51	N	3.0

*The table lists the van der Waals contacts and Hydrogen bonds observed from an atom in Protomer 1 (left of the table) to an atom in Protomer 2 (right of the table) in the loop 1 region. All contacts were calculated in the visualization software UCSF Chimera using the default parameters.

Supplementary Table 4. van der Waals contacts observed between the two protomers in L13MN at the QVVAG motif.

van der Waals Contacts at loop 1						
Atom in Protomer 1			Atom in Protomer 2			
Residue	Residue No.	Atom	Residue	Residue No.	Atom	Distance (Å)
GLY	52	C	ILE	47	CG1	3.6
ILE	47	CG1	GLY	52	C	3.6
ILE	47	CG1	GLY	52	O	3.3
GLY	52	CA	GLN	48	O	3.6
VAL	49	CG1	VAL	49	CG2	3.8
VAL	49	CG2	ALA	51	CB	4.0
ALA	51	CB	VAL	49	CG2	4.1
VAL	50	O	VAL	50	O	3.0
VAL	50	CA	VAL	50	O	3.6
GLN	48	O	ALA	51	CA	3.2
GLN	48	O	ALA	51	CB	3.4
ALA	51	CA	GLN	48	O	3.2
ALA	51	C	GLN	48	O	3.2
ALA	51	CB	GLN	48	O	3.4
ALA	51	CA	VAL	49	CA	3.4
GLN	48	CG	GLY	52	N	3.9
GLN	48	CG	GLY	52	CA	3.7
GLY	52	C	ILE	47	CG1	3.6
GLY	52	O	ILE	47	CG1	3.3

van der Waals Contacts at loop 3						
Atom in Protomer 1			Atom in Protomer 2			
Residue	Residue No.	Atom	Residue	Residue No.	Atom	Distance (Å)
VAL	74	CG2	VAL	74	CG2	3.4
VAL	75	O	VAL	74	CG2	3.3
VAL	74	CG2	VAL	75	O	3.3
VAL	75	C	VAL	74	CG2	3.7
VAL	74	CA	VAL	75	O	3.5
VAL	74	CG2	VAL	75	C	3.8
VAL	75	O	VAL	75	CG2	3.1
VAL	75	CG2	VAL	75	O	3.1
VAL	75	CG2	VAL	75	CG2	3.6
VAL	75	CG2	VAL	75	CG1	3.6
VAL	75	CG1	VAL	75	CG2	3.7
VAL	75	O	VAL	74	CA	3.4
VAL	75	CG2	VAL	75	CB	4.1
VAL	75	O	VAL	75	CA	3.7
VAL	75	CB	VAL	75	CG2	4.1
VAL	75	CA	VAL	75	O	3.7

van der Waals Contacts between loop 1 and loop 3

Atom in Protomer 1			Atom in Protomer 2			
Residue	Residue No.	Atom	Residue	Residue No.	Atom	Distance (Å)
VAL	74	CG1	GLN	48	NE2	3.3
GLN	73	CG	GLN	48	NE2	3.4
VAL	74	CG1	GLN	48	CG	3.8
GLN	73	CA	GLN	48	NE2	3.6
VAL	74	CA	GLN	48	NE2	3.7
VAL	74	CG1	GLN	48	CD	3.8
GLN	73	CB	GLN	48	NE2	3.9
VAL	74	CG1	VAL	50	CG2	3.8
VAL	74	CG2	VAL	50	CG1	3.6
VAL	74	O	GLN	48	NE2	3.2
VAL	74	CG1	VAL	50	CB	4.0
VAL	74	CG2	VAL	50	CB	3.8
VAL	74	N	GLN	48	NE2	2.9
GLN	48	NE2	GLN	73	CG	3.4
GLN	48	NE2	GLN	73	CA	3.6
GLN	48	CD	VAL	74	CG1	3.8
GLN	48	CG	VAL	74	CG1	3.8
GLN	48	NE2	GLN	73	CB	3.9
GLN	48	NE2	VAL	74	N	2.9
GLN	48	NE2	VAL	74	CG1	3.3
VAL	50	CG2	VAL	74	CG1	3.6
VAL	50	CG1	VAL	74	CG2	3.6
GLN	48	CG	VAL	74	CG1	3.8
GLN	48	NE2	VAL	74	O	3.2
GLN	48	NE2	VAL	74	CA	3.7
VAL	50	CB	VAL	74	CG1	4.0
VAL	50	CB	VAL	74	CG2	3.8

*The table lists the van der Waals contacts observed from an atom in Protomer 1 (left of the table) to an atom in Protomer 2 (right of the table) in the loop1 region, loop3 region, and in the region between loop1 and loop3 in the L13MN structure.

Supplementary Table 5. Hydrogen bonds observed between the two protomers at the QVVAG segments in L13MN

Hydrogen bonds at loop1

Atom in Protomer 1			Atom in Protomer 2			
Residue	Residue No.	Atom	Residue	Residue No.	Atom	Distance (Å)
VAL	50	N	VAL	50	O	2.9
VAL	50	O	VAL	50	N	2.9
GLN	48	O	GLY	52	N	2.9
GLY	52	N	GLN	48	O	2.6
GLY	52	O	ILE	47	N	3.0
ILE	47	N	GLY	52	O	3.2

Hydrogen bonds at loop3

Atom in Protomer 1			Atom in Protomer 2			
Residue	Residue No.	Atom	Residue	Residue No.	Atom	Distance (Å)
VAL	75	N	VAL	75	O	3.2
VAL	75	O	VAL	75	N	3.2
GLN	73	O	GLY	77	N	3.3
GLY	77	N	GLN	73	O	3.3

*The table lists the Hydrogen bonds observed from an atom in Protomer 1 (left of the table) to an atom in Protomer 2 (right of the table) in the loop1 region, and the loop3 region in the L13MN structure.

Supplementary Table 6. NMR and refinement statistics of the L1MK-Ctd dimer (PDB ID: 6IWJ)

	**L1MK-Ctd**
**Distance Restraints (NOE)**	
Intra (|i-j|=0)	480
Sequential (|i-j|=1)	384
Medium (|i-j| < 5)	238
Long (|i-j| => 5)	498
Total	1600
**Other Restraints**	
Hbonds	56
Dihedral Angles (ϕ,ψ)	204
**Rmsd[a] (Å)**	
All backbone atoms (Protomer A,B)	2.1
All heavy atoms	2.5
All backbone (Protomer A)	1.1
All backbone (Protomer B)	1.0
**RMS deviations**	
Bond Angles	1.3°
Bond Lengths	0.01Å
**Molprobity[b]**	
Clashscore	16.8
Cβ deviations > 0.25Å	0
Bad angles	0
Bad bonds	0
**Ramachandran Statistics[a]**	
Most Favoured regions (%)	87.3
Additionally Allowed regions (%)	12.3
Allowed regions (%)	0.4
Disallowed regions (%)	0.0

[a] Calculated for ordered residues of an ensemble of 20 lowest energy structures.
[b] Calculated for the lowest energy structure.

Supplementary Table 7. van der Waals contacts and hydrogen bonds observed between the two protomers in L1MK-Ctd at the QVVAG motif in loop1.

van der Waals contacts at loop1						
Atom in Protomer 1			Atom in Protomer 2			
Residue	Residue No.	Atom	Residue	Residue No.	Atom	Distance (Å)
ALA	16	O	VAL	12	CA	3.2
ALA	16	O	VAL	12	CB	3.5
VAL	14	CG2	ALA	16	O	3.5
VAL	12	CA	ALA	16	O	3.5
ALA	16	O	VAL	14	CG2	3.5
ALA	16	O	VAL	12	CG1	3.6
VAL	14	CG2	ALA	16	CB	3.7
ALA	16	O	VAL	12	C	3.8
GLY	17	CA	VAL	12	CA	3.9
VAL	12	C	ALA	16	O	3.9
ALA	16	C	VAL	12	CG1	3.9
VAL	14	CG1	VAL	14	CG2	4.0
ALA	16	CB	VAL	14	CG2	4.0
ALA	16	C	VAL	12	CA	4.0
VAL	14	CG2	ALA	16	C	4.0
VAL	16	N	ALA	14	CG2	4.0
ALA	16	C	VAL	12	CB	4.1
GLN	13	O	ALA	16	O	4.1
GLN	13	C	ALA	16	O	4.1
VAL	12	CB	ALA	16	O	4.2

Hydrogen Bonds at loop1						
Atom in Protomer 1			Atom in Protomer 2			
Residue	Residue No.	Atom	Residue	Residue No.	Atom	Distance (Å)
GLN	13	N	VAL	16	O	3.2
VAL	16	O	GLN	13	N	3.3

*The table lists the van der Waals contacts and Hydrogen bonds observed from an atom in Protomer 1 (left of the table) to an atom in Protomer 2 (right of the table) in the loop1 region.

Supplementary Table 8. Comparison of backbone hydrogen bonds observed between intermolecular β-sheets in engineered domain-swapped dimers with the respective intramolecular β-sheets in the monomeric wt proteins

**L1MN dimer**			**wt MNEI monomer**		
Donor (protomer 1)	Acceptor (protomer 2)	Distance (Å)	Donor (protomer 1)	Acceptor (protomer 1)	Distance (Å)
ASN 36 atom N	TYR 60 atom O	2.9	ASN 36 atom N	TYR 66 atom O	3.0
LYS 37 atom N	TYR 60 atom O	3.5	LYS 37 atom N	TYR 66 atom O	3.4
ILE 39 atom N	TYR 58 atom O	2.9	ILE 39 atom N	TYR 58 atom O	3.1
MET 43 atom N	GLN 56 atom O	3.0	MET 43 atom N	GLN 62 atom O	3.3
LYS 45 atom N	GLU 54 atom O	2.8	LYS 45 atom N	GLU 60 atom O	2.8
ILE 47 atom N	GLY 52 atom O	2.8	ILE 47 atom N	GLY 58 atom O	2.8
GLU 54 atom N	LYS 45 atom O	3.1	GLU 60 atom N	LYS 45 atom O	2.9
GLN 56 atom N	MET 43 atom O	2.9	GLN 62 atom N	MET 43 |atom O	2.8
TYR 60 atom N	LYS 37 atom O	2.8	TYR 66 atom N	LYS 37 atom O	2.8
SER 62 atom N	THR 34 atom O	3.0			

*The table lists the inter-protomer hydrogen bonds observed between β-strands 2 and 3 in L1MN dimer and the intramolecular hydrogen bonds observed between β-strands 2 and 3 in wt MNEI monomer. L1MN has a 6 amino acid deletion between β-strands 2 and 3 and the residue numbering for equivalent residues in LIMN and MNEI has a difference of 6 after the deletion.

**L1MK-Ctd dimer**			**wt MK-Ctd monomer**		
Donor (protomer 1)	Acceptor (protomer 2)	Distance (Å)	Donor (protomer 1)	Acceptor (protomer 1)	Distance (Å)
ARG 5 atom N	ILE 24 atom O	2.6	ARG 5 atom N	ILE 24 atom O	3.1
GLU 7 atom N	VAL 22 atom O	3.4	GLU 7 atom N	VAL 22 atom O	3.0
VAL 9 atom N	VAL 20 atom O	3.4	VAL 9 atom N	VAL 20 atom O	2.8
VAL 11 atom N	TRP 18 atom O	3.4	VAL 11 atom N	TRP 18 atom O	3.0
GLN 13 atom N	ALA 16 atom O	3.2	GLN 13 atom N	ALA 16 atom O	2.8
TRP 18 atom N	VAL 11 atom O	3.3	TRP 18 atom N	VAL 11 atom O	3.0
VAL 20 atom N	VAL 9 atom O	3.1	VAL 20 atom N	VAL 9 atom O	2.9
VAL 22 atom N	GLU 7 atom O	3.1	VAL 22 atom N	GLU 7 atom O	3.0
ILE 24 atom N	ARG 5 atom O	3.0	ILE 24 atom N	ARG 5 atom O	2.8
GLU 26 atom N	PRO 3 atom O	3.2	GLU 26 atom N	PRO 3 atom O	3.1

*The table lists the inter-protomer hydrogen bonds observed between β-strands 1 and 2 in L1MK-Ctd dimer and the intramolecular hydrogen bonds observed between β-strands 1 and 2 in wt MK-Ctd monomer.

Supplementary Methods

Purification of MNEI loop variants

MNEI and its variants were expressed in E. coli BL21 DE3* cells. A few colonies from the transformed cells were grown in Luria Bertani (LB) broth containing 100 µg mL^{-1} Ampicillin at 37°C, till an OD_{600} of 0.8 was reached. The starter culture was then diluted in Terrific Broth (1:10 ratio) and grown for 4-5 hours, followed by induction of protein expression by adding IPTG at a final concentration of 0.2 mM. The cells were grown for 12 hours after which they were pelleted (7000 RCF, 15-30 minutes, 4°C) and resuspended in 20 mM sodium acetate buffer, containing 1 mM EDTA, at pH 5.5 (Buffer A). The resuspended cells were sonicated at 60% amplitude for a total time of 30 minutes (5 sec ON, 2 sec OFF) on ice. Cell debris was removed by centrifugation (30,000 RCF, 45 minutes, 4°C). All subsequent steps were carried out in a cold room. Protein impurities were clarified by isoelectric precipitation; 300 mL buffer A was added to the total cell lysate and incubated for 2-3 hours, followed by centrifugation to remove the precipitate (30,000 RCF, 45 minutes, 4°C). The supernatant was loaded onto a SP-Sepharose cation exchange column (GE Healthcare), charged with 2 M NaCl, and equilibrated with Buffer A. This was followed by washing with ~20 column volumes (CV) of Buffer A. wt MNEI was eluted by step elution using Buffer A containing 100 mM NaCl (5 CV). Mutagenesis resulted in approximately a 0.2 to 0.8 pH unit increase in the isoelectric point for the different variants, hence, MNEI mutant variants were eluted using a linear gradient of 0 to 500 mM NaCl (5 CV) containing Buffer A. Different fractions were run on a 17.5% SDS-PAGE, and the fractions containing the protein were pooled, dialyzed against Milli-Q water and lyophilized. The molecular mass of each variant was confirmed by electrospray ionization mass spectrometry (ESI-MS). The protein concentration for each variant was estimated by using the molar extinction coefficient predicted by ExPASy[10].

For overexpression of $^{13}C/^{15}N$-labeled L3MN, *E. coli* BL21 DE3* cells were transformed with the expression vector, and a single colony was grown in 10 mL LB broth for 8 hours. 300 µL of the starter culture was used to inoculate 300 mL of M9 media containing ^{12}C glucose and ^{14}N NH_4Cl, and grown for 16 hours. The culture was then used to inoculate 6 L M9 minimal media containing ^{13}C glucose and ^{15}N NH_4Cl. Protein expression was induced at OD_{600} ~ 2 by adding IPTG at a final concentration of 0.4 mM. The cells were grown for 12-16 hours at 37°C. The protein was then purified following the protocol described above.

Purification of MK-Ctd loop variants

The C-terminal domain of MK0293 (PDB ID: 3C19, residues 98-178) was subcloned in pET-28a(+) vector to enable the expression of N-terminally 6xHis-tagged protein (with a thrombin protease cleavage site). The amino acid sequence of the protein is:

MGSSHHHHHHSSGLVPRGSHMVPSRFEDVRVVYGEREWRVSVKIAETEEGEVVTVKAEFDECREIGEETGIPPREVKAMVEAAARVGGWVDLKEREIKVQEG

The N-terminal tag residues are underlined (the numbering in the manuscript doesn't include the N-terminal tag). The His-tagged protein was purified by metal affinity chromatography using Ni^{2+}-NTA matrix. *E. coli* BL21 DE3* cells were grown after transformation with the expression vector and harvested, as described earlier for MNEI purification. Cells were grown in broth containing 50 μg mL^{-1} Kanamycin and protein expression was induced by adding IPTG at a final concentration of 0.2 mM. Cell pellet was resuspended in 20 mM Tris-HCl buffer, containing 150 mM NaCl, at pH 8 (Buffer B). The resuspended cells were sonicated, as described earlier, and cell debris was removed by centrifugation (30,000 RCF, 45 minutes, 4°C). All subsequent steps were carried out in a cold room. The clarified supernatant was incubated with 25 mL of Ni Sepharose 6 Fast Flow beads (GE Healthcare), charged with $NiSO_4$ and equilibrated with Buffer B. Binding was facilitated by gently rocking the mixture for 1-2 hours. The equilibrated mixture was loaded into a glass Econo-Column®Chromatography Column (Bio-Rad), and washed with 10 CV of Buffer B. This was followed by washing with 10 CV of Buffer B containing 5 mM imidazole and finally 5 CV of Buffer B containing 50 mM imidazole. The protein was eluted in 100 mL Buffer B containing 200 mM imidazole. 4 x 25 mL fractions were collected, which were run on a 17.5% SDS-PAGE. The fractions containing the protein were pooled, dialyzed against Milli-Q water and lyophilized. The same protocol was followed for the purification of MK-Ctd loop variants. The molecular mass of each protein was confirmed by ESI-MS. The protein concentration for each variant was estimated by using the molar extinction coefficient predicted by ExPASy[10]. To produce $^{13}C/^{15}N$-labeled MK-Ctd and L1MK-Ctd proteins, the clones were expressed as described for L3MN. The over-expressed proteins were then purified following the protocol described above.

Purification of Sso7dv loop variant

The sequence of wt Sso7d is given below. The positions that were randomized to create the Sso7d scaffold library and choose Sso7dv are shown in bold and underlined.

MATVKFKYKGEEKQVDISKI**KK**V**W**R**V**GK**M**I**S**F**T**YDLGGGK**T**G**R**G**A**VSEKDAPKEL LQKLEKQKK

Sso7dv and its variants were expressed in *E. coli* BL21 DE3* cells with an N-terminal 6xHis-SUMO (small ubiquitin-related modifier) expression tag, which was followed by a PreScission protease cleavage site. Protein expression was induced by adding IPTG at a final concentration of 1 mM, followed by growth for 12 hours at 37°C. The cells were harvested, resuspended in lysis buffer (50 mM Tris-HCl buffer, containing 10 mM imidazole and 500 mM NaCl, at pH 7.5) and sonicated. Soluble protein in the clarified supernatant, post sonication, was purified by metal affinity chromatography using Ni^{2+}-NTA matrix, as described earlier for MK-Ctd purification. The protein was eluted using a linear gradient of 100 to 500 mM imidazole. The eluate was further purified by gel filtration chromatography on HiLoad® 16/600 Superdex® 75pg column (GE Healthcare), equilibrated with 50 mM Tris-HCl buffer, containing 250 mM NaCl, pH 7.5. The N-terminal 6xHis-SUMO tag was removed from the purified fusion protein using PreScission protease. Protease digestion was carried out at 4°C for 14-16 hours, followed by separation of Sso7dv (or its variants) from the cleaved tag using gel filtration chromatography on HiLoad® 16/600 Superdex® 75pg column (GE Healthcare), equilibrated with 50 mM phosphate buffer, containing 200 mM NaCl, pH 7.5. Purified protein was stored at 4°C in the presence of 0.03% (wt/vol) sodium azide.

Purification of UBQ loop variants

Wild type UBQ and L1UBQ were purified from the soluble fraction of the cell lysate. Briefly, the proteins were expressed in *E. coli* BL21 DE3* cells, as described earlier for MNEI. Protein expression was induced by adding IPTG at a final concentration of 0.35 mM. Cell pellet was resuspended in 50 mM sodium acetate buffer, containing 5 mM EDTA, pH 4.5-4.75 (Buffer C). Cells were sonicated, as described before, and the cell debris was removed by centrifugation. The pH of the supernatant was adjusted to 4.5-4.75 by adding dilute acetic acid, with continuous stirring. The precipitate formed was removed by passing the supernatant through a 0.45 μm syringe filter. The supernatant was loaded onto a SP-Sepharose cation exchange column (GE Healthcare), charged with 2 M NaCl, and equilibrated with Buffer C. After washing the column with ~20 CV of Buffer C, the protein was eluted with a continuous salt gradient of 0 to 600 mM NaCl in Buffer C, within 5 CV. Different fractions were run on a 17.5% SDS-PAGE, and the fractions containing the protein were pooled, dialyzed against Milli-Q water and lyophilized. The purification of L1UBQ was carried out at pH 3.7 (instead of pH 4.5-4.7 used for wt UBQ), due to a decrease in the

isoelectric point of L1UBQ by almost a pH unit upon mutagenesis. The molecular mass of each protein was confirmed by ESI-MS. The protein concentration for each variant was estimated by using the molar extinction coefficient predicted by ExPASy[10]. Unfolding-refolding of purified UBQ and L1UBQ was carried out by incubating the proteins at ~ 1 mM concentration in 8 M urea, 20 mM Tris-HCl, pH 8.3 for 2-3 hours at RT. This was followed by removal of urea using the same dialysis protocol that is described below for L4UBQ, and the final urea concentration was maintained at 0.4 M.

L4UBQ and L4UBQ* were purified from the insoluble fraction of the cell lysate. Briefly, bacterial cells over-expressing the L4UBQ protein were pelleted and resuspended in 50 mM sodium acetate buffer, containing 5 mM EDTA, pH 4. The cells were lysed by sonication on ice (for a total time of 30 minutes at 60% amplitude on ice; 5 sec ON, 2 sec OFF) and the supernatant was discarded after centrifugation. The pellet was resuspended in ~120 mL of 8 M urea, 20 mM Tris-HCl, pH 8.3, followed by sonication (using the same settings) for 15 minutes on ice and centrifugation. The supernatant was collected and the pellet was resuspended again in ~100 mL of urea-buffer, followed by another round of sonication for 15 minutes on ice and centrifugation. The supernatants from the two rounds of sonication were pooled (~ 200 mL) and dialyzed against (4 L) 20 mM Tris-HCl, pH 8.3 for 6 hours at 4°C. This was followed by another round of dialysis against 4 L of 20 mM Tris-HCl, pH 8.3, containing 0.4 M urea, for 6-8 hours at 4°C. The final urea concentration was maintained at 0.4 M in both the rounds of dialysis. Removal of urea resulted in precipitation; the precipitate was removed by centrifugation at 30,000 RCF for 30 minutes. The pH of the clarified supernatant was adjusted to 8.3 and the refolded protein was purified using anion-exchange chromatography in the presence of 0.4 M urea. The supernatant was loaded on 2x5 mL HiTrap™ DEAE FF columns (GE Healthcare), followed by washing till a stable baseline was achieved. The protein was finally eluted using a linear gradient of 0 to 1 M NaCl. Different fractions were collected and run on a 17.5 % SDS-PAGE, L4UBQ was found to elute between 0.6 to 1 M NaCl. The fractions containing the protein were pooled and dialyzed against 20 mM Tris-HCl, 5 mM EDTA, pH 8.3. The dialyzed protein was stored at 4°C in the presence of 0.03% (wt/vol) sodium azide. L4UBQ** was purified from the soluble fraction of the cell lysate at pH 4.5, similar to wt UBQ, because the protein was found to precipitate when the pH was lowered to ~3.7. The molecular mass of each variant was confirmed by ESI-MS. The protein concentration for each variant was estimated by using the molar extinction coefficient predicted by ExPASy[10].

Multi-angle light scattering

Absolute molar mass calculations were done using light scattering measurements carried out on a DAWN 8+, eight angle light scattering instrument (Wyatt Technology Corp., Santa Barbara, CA). The concentration of the dimeric fraction for the different MNEI, UBQ and MK-Ctd loop variants, and the void fraction for L4UBQ, isolated from size exclusion chromatography, was adjusted to about 1.5 mg ml^{-1}. Proteins were run through a 0.02 μm filter into the light scattering fused silica flow cell at a constant rate. Scattering intensity was normalized using a solution of monomeric bovine serum albumin. Data analysis was done using the software Astra.

Thioflavin T binding

ThT binding to wt UBQ, void/oligomeric fraction of L4UBQ (separated on SEC) and amyloid fibrils of Tau K18[3] was carried out by mixing the protein and ThT to achieve a final protein concentration of 1 μM and a final ThT concentration of 10 μM in 20 mM Tris-HCl buffer containing 150 mM NaCl, at pH 8.3. The fluorescence emission spectra of the mixtures and ThT alone were acquired using a FluoroMax-3 (Horiba JobinYvon) spectrofluorimeter. The samples were excited at 440 nm, with an excitation bandwidth of 1 nm, and the emission was monitored from 450 to 550 nm, with an emission bandwidth of 10 nm. A signal averaging time of 0.5 s was used and three scans were averaged for each sample. The path length of the cuvette used was 10 mm.

Equilibrium unfolding experiments

Equilibrium unfolding experiments, where the circular dichroism signal at 222 nm of the four proteins was monitored, were carried out using a protein concentration of 10-20 μM. The experiments were carried out in 50 mM phosphate buffer, pH 7. The data were acquired on a Jasco J-815 spectropolarimeter in a 1 mm path length quartz cuvette. The proteins were incubated with different concentrations of GdnHCl for 2-6 h at 25°C before making the measurement. The concentration of GdnHCl in unfolding buffer was determined by refractive index measurement.

Molecular dynamics (MD) simulations

MD simulations of symmetrized structure-based models of L2MN and L3MN were performed to obtain simulated structures of the L2MN and L3MN domain-swapped dimers. Details of the simulation model and protocol are given elsewhere[11]. Briefly, monomer structures of L2MN and L3MN were built from the structure of single chain monellin (PDB

ID: 1IV7; chain A) by mutating loop2 residues (^{65}YASDK^{69}) and loop3 residues (^{78}DYKTR^{82}) respectively to QVVAG. All heavy atom structure-based models[12] (SBMs) of L2MN and L3MN monomers were then created from these structures. The minima of the dihedral potentials of either loop2 (for L2MN) or loop3 (for L3MN) were then set to the dihedral angles of the ^{46}QVVAG^{50} residues present in the dimer structure of stefin-B[13] (PDB ID: 2OCT, chain A). These single chain SBMs were then used to create a symmetrized SBM by including two identical protein chains, say, X and Y. Further, every contact within the chains ((i_X, j_X) and (i_Y, j_Y)) was also supplemented with contacts (i_X, j_Y) and (i_Y, j_X) between the chains. This symmetrized model was then simulated using MD simulations. Representative domain-swapped structures from these simulations with either loop2 or loop3 swapped were then chosen to create the simulated SAXS profiles.

References

1. Shen, Y., Delaglio, F., Cornilescu, G. & Bax, A. TALOS+: a hybrid method for predicting protein backbone torsion angles from NMR chemical shifts. *J. Biomol. NMR* **44,** 213–223 (2009).
2. Stott, K., Blackburn, J. M., Butler, P. J. & Perutz, M. Incorporation of glutamine repeats makes protein oligomerize: implications for neurodegenerative diseases. *Proc. Natl. Acad. Sci.* **92,** 6509–6513 (1995).
3. Ramachandran, G. & Udgaonkar, J. B. Evidence for the existence of a secondary pathway for fibril growth during the aggregation of tau. *J. Mol. Biol.* **421,** 296–314 (2012).
4. Agashe, V. R. & Udgaonkar, J. B. Thermodynamics of denaturation of barstar: evidence for cold denaturation and evaluation of the interaction with guanidine hydrochloride. *Biochemistry* **34,** 3286–3299 (1995).
5. Patra, A. K. & Udgaonkar, J. B. Characterization of the folding and unfolding reactions of single-chain monellin: evidence for multiple intermediates and competing pathways. *Biochemistry* **46,** 11727–43 (2007).
6. Gera, N., Hussain, M., Wright, R. C. & Rao, B. M. Highly stable binding proteins derived from the hyperthermophilic Sso7d scaffold. *J. Mol. Biol.* **409,** 601–616 (2011).
7. Went, H. M. & Jackson, S. E. Ubiquitin folds through a highly polarized transition state. *Protein Eng. Des. Sel.* **18,** 229–237 (2005).

8. Khorasanizadeh, S., Peters, I. D., Butt, T. R. & Roder, H. Folding and stability of a tryptophan-containing mutant of ubiquitin. *Biochemistry* **32,** 7054–7063 (1993).

9. Chen, V. B. *et al.* MolProbity: all-atom structure validation for macromolecular crystallography. *Acta Crystallogr. Sect. D Biol. Crystallogr.* **66,** 12–21 (2010).

10. Gasteiger, E. *et al. Protein identification and analysis tools on the ExPASy server*. (Springer, 2005).

11. Mascarenhas, N. M. & Gosavi, S. Understanding protein domain-swapping using structure-based models of protein folding. *Prog. Biophys. Mol. Biol.* (2016).

12. Whitford, P. C. *et al.* An all- atom structure- based potential for proteins: Bridging minimal models with all- atom empirical forcefields. *Proteins Struct. Funct. Bioinforma.* **75,** 430–441 (2009).

13. Mascarenhas, N. M. & Gosavi, S. Protein Domain-Swapping Can Be a Consequence of Functional Residues. *J. Phys. Chem. B* **120,** 6929–6938 (2016).
