# Supplementary material for: A five-residue motif for the design of domain swapping in proteins
Source: Nat Commun. 2019 Jan 28;10:452. doi: 10.1038/s41467-019-08295-x (PMC6349918; doi:10.1038/s41467-019-08295-x)
Supplement: Supplementary file 2 — Reporting Summary [file 41467_2019_8295_MOESM2_ESM.pdf]

nature research

Corresponding author(s): SHACHI GOSAVI

Last updated by author(s): Dec 17, 2018

Reporting Summary

Nature Research wishes to improve the reproducibility of the work that we publish. This form provides structure for consistency and transparency in reporting. For further information on Nature Research policies, see Authors & Referees and the Editorial Policy Checklist.

Statistics

For all statistical analyses, confirm that the following items are present in the figure legend, table legend, main text, or Methods section.

n/a	Confirmed	
☐	☒	The exact sample size (*n*) for each experimental group/condition, given as a discrete number and unit of measurement
☐	☒	A statement on whether measurements were taken from distinct samples or whether the same sample was measured repeatedly
☒	☐	The statistical test(s) used AND whether they are one- or two-sided<br>*Only common tests should be described solely by name; describe more complex techniques in the Methods section.*
☒	☐	A description of all covariates tested
☒	☐	A description of any assumptions or corrections, such as tests of normality and adjustment for multiple comparisons
☐	☒	A full description of the statistical parameters including central tendency (e.g. means) or other basic estimates (e.g. regression coefficient) AND variation (e.g. standard deviation) or associated estimates of uncertainty (e.g. confidence intervals)
☒	☐	For null hypothesis testing, the test statistic (e.g. *F*, *t*, *r*) with confidence intervals, effect sizes, degrees of freedom and *P* value noted<br>*Give P values as exact values whenever suitable.*
☒	☐	For Bayesian analysis, information on the choice of priors and Markov chain Monte Carlo settings
☒	☐	For hierarchical and complex designs, identification of the appropriate level for tests and full reporting of outcomes
☒	☐	Estimates of effect sizes (e.g. Cohen's *d*, Pearson's *r*), indicating how they were calculated

Our web collection on statistics for biologists contains articles on many of the points above.

Software and code

Policy information about availability of computer code

Data collection	Chromatography data from AKTA FPLC was collected using UNICORN 5.11; Fluorescence data from FluoroMax-3 was collected using DataMax ver. 2.2; Circular Dichroism data was collected using JASCO Spectra Manager Suite; MALS data was collected using Astra; Mass spectra were collected using MassLynx V4.1; protein and ThT concentrations were estimated by using Cary UV-Vis spectrophotometer and the data was collected using Cary WinUV software; NMR data was collected using Topspin; SAXS data was collected using ATSAS; X-ray diffraction data was collected using HKL3000; Cryo-EM images were acquired using TIA software.
Data analysis	Size-exclusion profiles, equilibrium unfolding transitions, CD and ThT data were analyzed using SigmaPlot version 12.0, from Systat Software, Inc., San Jose California; NMR data was processed and analyzed using the NMRPipe, Sparky and TALOS+ softwares; X-ray diffraction data was processed and analyzed using the XDS, POINTLESS, AIMLESS, PHENIX, COOT and MOLPROBITY softwares; SAXS data was processed and analyzed suing the FoXS, Primus and Gnom softwares; MALS data was analyzed using Astra.

For manuscripts utilizing custom algorithms or software that are central to the research but not yet described in published literature, software must be made available to editors/reviewers. We strongly encourage code deposition in a community repository (e.g. GitHub). See the Nature Research guidelines for submitting code & software for further information.

Data

Policy information about availability of data

All manuscripts must include a data availability statement. This statement should provide the following information, where applicable:

- Accession codes, unique identifiers, or web links for publicly available datasets
- A list of figures that have associated raw data
- A description of any restrictions on data availability

The coordinates and structure factors for L1MN, L13MN, and L3MN monomer have been deposited in the Protein Data Bank (PDB), under the accession codes 5YCU [http://dx.doi.org/10.2210/pdb5YCU/pdb], 5YCW [http://dx.doi.org/10.2210/pdb5YCW/pdb] and 5YCT [http://dx.doi.org/10.2210/pdb5YCT/pdb], respectively. The coordinates of the NMR models of the L1MK-Ctd dimer have been deposited in the PDB, under the accession code 6IWJ. NMR data for the L3MN monomer, L3MN dimer and L1MK-Ctd dimer are deposited in the BMRB under the accession codes 27248, 27247 and 36222, respectively. The source data underlying Figures 2a, 2b,

2c, 3a, 3b, 3d, 3e, 3f, 4a, 5b, 5d, 6b, 6d, and Supplementary Figures 4, 5, 9, 13, 16, 17, 19, 20, 21a, 21c and 22 are provided as a Source Data file, available as a Supplementary Information file. A reporting summary for this Article is available as a Supplementary Information file. All unique materials are available on reasonable request from the corresponding authors.

Field-specific reporting

Please select the one below that is the best fit for your research. If you are not sure, read the appropriate sections before making your selection.

☒ Life sciences ☐ Behavioural & social sciences ☐ Ecological, evolutionary & environmental sciences

For a reference copy of the document with all sections, see nature.com/documents/nr-reporting-summary-flat.pdf

Life sciences study design

All studies must disclose on these points even when the disclosure is negative.

Sample size	Not relevant to our study
Data exclusions	No data were excluded
Replication	Noted in text
Randomization	Not relevant to our study; no in vivo studies were carried out
Blinding	Not relevant to our study; no in vivo studies were carried out

Reporting for specific materials, systems and methods

We require information from authors about some types of materials, experimental systems and methods used in many studies. Here, indicate whether each material, system or method listed is relevant to your study. If you are not sure if a list item applies to your research, read the appropriate section before selecting a response.

Materials & experimental systems

n/a	Involved in the study	
☒	☐	Antibodies
☒	☐	Eukaryotic cell lines
☒	☐	Palaeontology
☒	☐	Animals and other organisms
☒	☐	Human research participants
☒	☐	Clinical data

Methods

n/a	Involved in the study	
☒	☐	ChIP-seq
☒	☐	Flow cytometry
☒	☐	MRI-based neuroimaging
